# Differential recruitment drives pathogen‐mediated competition between species in an amphibian chytridiomycosis system

**DOI:** 10.1002/eap.3085

**Published:** 2025-01-17

**Authors:** Madelyn J. Mangan, Hamish I. McCallum, Matt West, Ben C. Scheele, Graeme R. Gillespie, Laura F. Grogan

**Affiliations:** ^1^ Centre for Planetary Health and Food Security, and School of Environment and Science Griffith University Southport Queensland Australia; ^2^ School of BioSciences University of Melbourne Parkville Victoria Australia; ^3^ Wild Research Pty Ltd Warrandyte Victoria Australia; ^4^ Fenner School of Environment and Society The Australian National University Canberra Australian Capital Territory Australia; ^5^ School of the Environment The University of Queensland Brisbane Queensland Australia

**Keywords:** amphibian, amplifying host, *Batrachochytrium dendrobatidis*, elevation, interactive threats, non‐native predator, pathogen‐mediated apparent competition, recruitment, reservoir host

## Abstract

Pathogens that infect multiple host species have an increased capacity to cause extinctions through parasite‐mediated apparent competition. Given unprecedented and continuing losses of biodiversity due to *Batrachochytrium dendrobatidis* (Bd), the causative fungus of the amphibian skin disease chytridiomycosis, a robust understanding of the mechanisms driving cross‐species infection dynamics is essential. Here, we used stage‐structured, susceptible‐infected compartmental models to explore drivers of Bd‐mediated apparent competition between two sympatric amphibians, the critically endangered *Litoria spenceri* and the non‐threatened *Litoria lesueurii*. We additionally simulated the impact of plausible *L. spenceri* conservation management interventions on competitive outcomes between these two species. Despite being more susceptible to disease than its competitor, a high relative rate of recruitment allowed the non‐threatened *L. lesueurii* to reach substantially higher densities than *L. spenceri* in our baseline models, applying a strong absolute force of infection on *L. spenceri* as an amplifying host. However, simulated management interventions which bolstered *L. spenceri* recruitment (i.e., captive breeding and release, removal of predatory non‐native trout) spurred strong recoveries of *L. spenceri* while simultaneously (1) increasing the force of Bd infection in the environment and (2) reducing *L. lesueurii* population density. At high and moderate elevations, combined captive breeding/release and non‐native trout removal were sufficient to make *L. spenceri* the most abundant species. Overall, our results demonstrate the importance of recruitment in moderating pathogen dynamics of multi‐host amphibian chytridiomycosis systems. While infection‐based parameters are undoubtedly important in Bd management, modifying relative rates of recruitment can substantially alter pathogen‐mediated competition between species of an amphibian community.

## INTRODUCTION

Pathogens are increasingly recognized as an important threat to wildlife populations (Daszak et al., 2000; Fisher et al., 2012; Jones et al., 2008). In particular, the fungus *Batrachochytrium dendrobatids* (Bd) has caused a greater loss of vertebrate species than any pathogen in recorded history (Skerratt et al., 2007). Bd is the causative agent of the amphibian skin disease chytridiomycosis, which has contributed to the presumed extinctions of at least 90 species and continues to threaten vulnerable taxa in the Americas, Europe, Africa, and Oceania (Scheele et al., 2019). Bd zoospores encyst on keratinized skin of amphibians where they form sporangia and propagate within host cells, frequently causing fatal disruption of skin function, osmoregulation, and metabolism as infection progresses (Grogan et al., 2018, 2020; Ohmer et al., 2017; Voyles et al., 2009). The fungus is transmitted within and between amphibian species via motile Bd zoospores which are shed by infected individuals into shared waterways and habitat, or through direct contact between individuals (Berger et al., 2005; Burns et al., 2021; Kilpatrick et al., 2010; Longcore et al., 1999). Bd is difficult to eradicate once established in amphibian communities and can maintain off‐host infectivity for long periods of time in the environment (Johnson & Speare, 2003; Scheele et al., 2014; Skerratt et al., 2016).

Many infectious diseases impact biodiversity; however, multi‐host pathogens such as Bd carry increased risks of causing host extinction due to pathogen‐mediated apparent competition (De Castro & Bolker, 2005). Apparent competition occurs when competitive interactions between species are mediated through a common enemy (e.g., a predator, parasitoid, or pathogen) as opposed to direct competition between species (Holt & Lawton, 1994). Theoretically, single‐host pathogens with transmission dependent on host density should self‐extinguish before causing species extinction. However, persistent transmission of a generalist pathogen from other host populations can drive continued decline and extinction of a species even as it reaches low densities (De Castro & Bolker, 2005; Hudson & Greenman, 1998; McCallum, 2012).

Across diverse multi‐host parasite systems, apparent competition is especially salient when there is heterogeneity in infection parameters (e.g., susceptibility to disease, transmission, and shedding rate) among groups within a shared transmission network. For example, differential susceptibility to infection or disease can transform baseline competitive outcomes by asymmetrically increasing mortality or depressing rates of recruitment across species (Kiesecker & Blaustein, 1999; Parris & Cornelius, 2004; Rushton et al., 2000; Schall, 1992; Schmitz & Nudds, 1994). Heterogeneity in transmission or shedding rates can further fuel pathogen‐mediated competition by allowing a single species to act as an amplification host, disproportionally driving increases in the pathogen reproductive rate (*R*_0_) across an entire host community (Cobb et al., 2010; DiRenzo et al., 2014; Kilpatrick et al., 2006; Power & Mitchell, 2004).

In the case of Bd, variability in infection tolerance across species, populations, and life stages is suspected to drive many amphibian declines and extinctions via pathogen‐mediated competition (Bradley et al., 2015; Briggs et al., 2010; Gervasi et al., 2013; Medina et al., 2015; Scheele, Hunter, et al., 2017; Scheele, Skerratt, et al., 2017). In the absence of complementary resistance mechanisms to reduce pathogen burden, hosts that are more tolerant (i.e., able to limit morbidity and mortality from a given pathogen load) tend to amplify the concentration of Bd in amphibian systems (Grogan et al., 2023). In specific contexts, tolerant hosts may even act as reservoirs—meaning they facilitate continued transmission to susceptible populations which cannot independently maintain a pathogen (Grogan et al., 2023; Haydon et al., 2002; McCallum, 2012; Roberts & Heesterbeek, 2020). Such tolerance‐based competitive exclusion is evidenced in several amphibian communities. In Australia, the critically endangered and Bd‐susceptible corroboree frog (*Pseudophryne pengilleyi*) shows 41.4% prevalence of infection when sympatric with the tolerant host *Crinia signifera*, but only 2.6% prevalence when *C. signifera* is absent (Scheele, Hunter, et al., 2017). In the Southwestern United States and Sonora, Mexico, the presence of the highly Bd‐tolerant and invasive American bullfrog (*Lithobates catesbeianus*) is associated with both increased occurrence of Bd and ranavirus as well as decreased occurrence of native amphibians (Hossack, Oja, et al., 2023). Furthermore, *L. catesbeianus* was experimentally indicated as a reservoir host in Arizona sites when its removal resulted in complete extirpation of Bd and ranavirus from the amphibian communities it once occupied (Hossack, Hall, et al., 2023).

However, while infection‐based characteristics such as tolerance undoubtedly contribute to pathogen amplification in amphibian communities, competitive outcomes of chytridiomycosis systems are sometimes contrary to predictions based on infection parameters alone. In recent decades, the critically endangered spotted tree frog (*Litoria spenceri*) has undergone extensive declines throughout its range in southeast Australia, largely due to the combined impacts of Bd infection and predation by introduced trout (Gillespie, 2001; Gillespie et al., 2015; West et al., 2024). Meanwhile, the sympatric and non‐threatened Lesueur's frog (*L. lesueurii*) shows no evidence of decline despite being *more susceptible* to infection and disease. Indeed, multistate mark‐recapture modeling from six years of monitoring data in wild populations reveals that the non‐declining *L. lesueurii* displays substantially higher Bd infection rates and over twice the annual rate of Bd‐associated mortality than the critically endangered *L. spenceri* (West, 2015).

Given worse infection outcomes in the non‐declining species *L. lesueurii*, it has been proposed that a relatively high rate of recruitment allows *L. lesueurii* to compensate for Bd‐associated mortality, ultimately acting as a Bd reservoir host which further exacerbates *L. spenceri* declines (West et al., 2020). *Litoria lesueurii* matures roughly a year faster and typically produces a clutch size over two times larger than *L. spenceri* on average (*L. spenceri* clutch size: 477–720, *L. lesueurii* clutch size: 1400–1602; Gillespie, 2010, 2011a). Additionally, non‐native trout (*Salmo trutta* and *Oncorhynchus mykiss*) substantially predate *L. spenceri* larvae but not those of the non‐threatened *L. lesueurii* (Gillespie, 2001). Together, these factors cause severe asymmetry in recruitment rates between the two species, which may bolster the role of *L. lesueurii* as an amplifying host while impeding the capacity of *L. spenceri* to buffer against Bd‐associated mortality. This hypothesis was supported by observational evidence in multispecies dynamic occupancy models, which showed that *L. spenceri* extinction risk increases at sites where *L. lesueurii* is also present (West et al., 2024).

West et al. (2020) used discrete‐time population matrix models to investigate this system. They concluded that differences in vital rates between the two species were critically important in enabling *L. lesueurii* to persist in the presence of Bd, whereas *L. spenceri* could not. However, they assumed constant rates of Bd infection and recovery for each species. This approach therefore did not include epidemiological dynamics and in particular could not account for cross‐species infection dynamics which are integral in shaping epidemiological outcomes of multi‐host amphibian communities (Wilber et al., 2020). This meant that the hypothesis that *L. lesueurii* exacerbates *L. spenceri* declines through pathogen‐mediated competition was not evaluated mechanistically. Extending the work of West et al. (2020) to explicitly incorporate host pathogen dynamics is the key objective of our study.

Here, we investigate pathogen‐mediated competitive dynamics of the *L. spenceri/L. lesueurii* system, an amphibian community where population outcomes contradict the observed differential in susceptibility to Bd between species. We used stage‐structured, two‐species susceptible‐infected compartmental modeling to elucidate what factors moderate baseline competitive outcomes in this system and identify management interventions that most effectively foster recoveries of the critically endangered *L. spenceri*. Specifically, we simulated (1) the targeted removal of the suspected amplification host *L. lesueurii*, (2) removal of non‐native trout, and (3) supplementation of the *L. spenceri* larval population through captive breeding and release. Our compartmental models explicitly incorporate cross‐species transmission and dynamic infection rates to investigate how interactions of disease, predation, and demography shape competitive outcomes in a two‐species amphibian system. Beyond simply evaluating leverage of prospective *L. spenceri* management interventions, this approach highlights the importance of community context in moderating the many synergistic threats which amphibian species face around the globe.

## METHODS

### Model structure

We created continuous‐time compartmental models for Bd dynamics in the *L. spenceri*/*L. lesueurii* system using field‐based parameter estimates for low‐, moderate‐, and high‐elevation sites as described in West et al. (2020). Our models represent a small number of mountain streams in northeastern Victoria and southern New South Wales in Australia where there is sympatry between the critically endangered *L. spenceri* and the more widely distributed, non‐threatened *L. lesueurii*. These species co‐occur at some (but not all) *L. spenceri* localities, which range from 335 m above sea level (January–February daily avg. max. temperature 25–27°C, July daily avg. min. 8–9°C) to 1100 m (January–February daily avg. max. temperature 20–24°C, July daily avg. min. −1.5°C; Gillespie, 2011b). Breeding for both species occurs primarily in November and December across elevations, and metamorphosis is completed within three months of hatching—usually by March or April (Hero et al., 1995).

**FIGURE 1:**
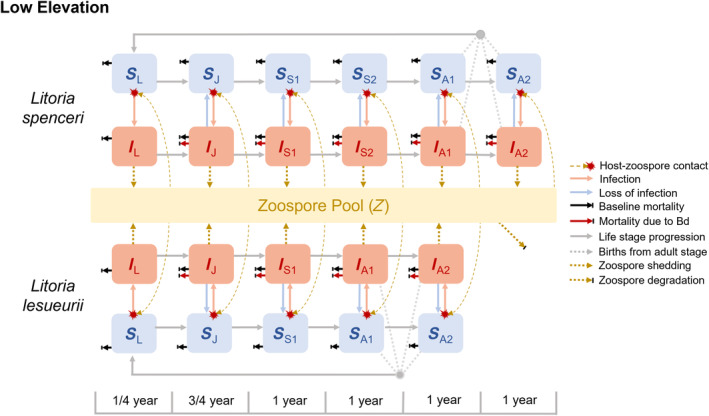
Two‐host susceptible‐infected model structure for *Litoria spenceri* and *Litoria lesueurii* at low elevations. Susceptible hosts (*S*, blue) acquire infection from a common zoospore pool (*Z*, gold) to transition to the Infected state (*I*, red). Infected hosts shed zoospores into the pool until they either (1) die from infection or natural causes or (2) lose infection and return to the susceptible state. Host‐zoospore contact rate is not explicitly parameterized in the model; it is an assumed component of the transmission rate. Each species ages sequentially through larval, juvenile, subadult, and adult life stages. Life stage progression requires an average of one year for all stages except the larva‐to‐juvenile (3 months) and juvenile‐to‐subadult (9 months) transitions. Across elevations, *L. lesueurii* requires one year less on average in the subadult stage. Compared with the low elevation model pictured here, both species also require one additional year in the subadult stages at moderate elevations and two additional years at high elevations. Parameter values corresponding to each life stage and species are in Table 1.

Each of our three compartmental models is a two‐host, multistage susceptible‐infected model where all pathogen transmission occurs through a dynamic zoospore pool rather than by direct transmission (Figure 1). Model outputs were generated in R v4.4.1 using the package “deSolve” (Soetaert et al., 2010). Amphibian hosts in the models progress through up to eight life stages depending on species and elevation, spending an average of three months in the larval stage (L), nine months in the juvenile stage (J), one year in each subadult stage (S1, S2, S3, S4), and one year in the first adult stage (A1) before entering the second adult stage (A2) until mortality. Given colder temperatures at high elevation sites, both species mature more quickly at lower elevations such that lower elevations have fewer subadult stages (Gillespie, 2011b). *Litoria lesueurii* females additionally tend to mature one year earlier (2–4 years) than *L. spenceri* females (3–5 years) across all elevations (Gillespie, 2011b; West et al., 2020). Thus, the models representing low‐ (~350 m), moderate‐ (~735 m), and high‐elevation (~1100 m) populations contain five, six, and seven total stages for *L. lesueurii* and six, seven, and eight stages for *L. spenceri*, respectively (Figure 1).

Given a life stage k, the parameter definitions are

1. $\alpha_{k}\left(t\right),$ seasonally forced per capita birth rate for offspring of life stage k;
2. $S_{k},$ density of susceptible individuals in life stage k;
3. $I_{k},$ density of infected individuals in life stage k;
4. $N_{k},$ density of all individuals in life stage k;
5. $\beta_{k},$ rate of transmission from the zoospore pool to life stage k;
6. $m_{k},$ baseline mortality rate of life stage k;
7. $\mu_{k},$ added mortality rate due to Bd in life stage k;
8. $\gamma_{k},$ rate of infection loss in life stage k;
9. $\delta_{k},$ rate of aging from life stage k to the next life stage;
10. $\lambda_{k},$ zoospore shedding rate from infected individuals in life stage k;
11. $\gamma_{z},$ off‐host zoospore decay rate.
12. Z, density of zoospores in the zoospore pool;
13. $Z_{\min},$ minimum ambient density of zoospores (from other sources).

For either species, in each life stage k, the generalized differential equations are

(1) $$\frac{{\mathrm{dS}}_{k}}{\mathrm{dt}}=\delta_{k-1}S_{k-1}-\beta_{k}S_{k}Z-m_{k}S_{k}+\gamma_{k}I_{k}-\delta_{k}S_{k},$$

(2) $$\frac{{\mathrm{dI}}_{k}}{\mathrm{dt}}=\delta_{k-1}I_{k-1}+\beta_{k}S_{k}Z-m_{k}I_{k}-\mu_{k}I_{k}-\gamma_{k}I_{k}-\delta_{k}I_{k}.$$

An exception to this format accommodates births into the susceptible compartment of the larval stage using the seasonally forced birth rate $\alpha_{k}\left(t\right)$ and the larval carrying capacity $K_{L}$. Differential equations for the larval stage are

(3) $$\begin{array}{c} \frac{{\mathrm{dS}}_{L}}{\mathrm{dt}}=\left(1-\frac{N_{L}}{K_{L}}\right)\left(\alpha_{A1}\left(t\right)\times N_{A1}+\alpha_{A2}\left(t\right)\times N_{A2}\right)-\beta_{L}S_{L}Z-m_{L}S_{L}+\gamma_{L}I_{L}-\delta_{L}S_{L}, \end{array}$$

(4) $$\frac{{\mathrm{dI}}_{L}}{\mathrm{dt}}=\beta_{L}S_{L_{1}}Z-m_{L}I_{L}-\gamma_{L}I_{L}-\delta_{L}I_{L}.$$

The differential equation for the dynamic zoospore pool from which infection is transmitted can be expressed as the summation of zoospore decay and the zoospores shed from infected stages across both species (where *h* represents each species). An additional term incorporating the minimum ambient density of zoospores $Z_{\min}$ prevents unrealistic pathogen extinction during invasion, as shown below:

(5) $$\begin{array}{c} \frac{\mathrm{dZ}}{\mathrm{dt}}=\sum_{h,k}\lambda_{k}I_{h,k}-\gamma_{Z}Z+\gamma_{Z}Z_{\min}. \end{array}$$

### Model parameterization

#### General parameters

Finite annual rates from discrete models in West et al. (2020) were converted to instantaneous daily rates to parameterize our model (Table 1). These original parameter estimations were derived from long‐term mark‐recapture studies (M. West & G. Gillespie, unpublished data), counts of egg clutches (Gillespie, 2011a), and skeletochronology (Gillespie, 2010). Species differences in susceptibility to disease (added mortality due to Bd infection) in West et al. (2020) were estimated from Bayesian multistate mark‐recapture modeling with six years of surveys in wild populations, using capture data from 295 unique *L. spenceri* adults and 850 unique *L. lesueurii* adults (West, 2015). The temporal resolution of field data was insufficient to separately parameterize mortality for larvae and juveniles, so we assigned their joint baseline mortality rate to both stages in our model. We also assumed that juveniles exhibit the same added mortality due to Bd (μ) as first year subadults and that larvae neither die from infection nor recover from infection. While larval Bd infection is common across amphibian species and frequently impacts developmental rates, direct Bd‐associated mortality is rare prior to metamorphosis (Blaustein et al., 2005; Davidson et al., 2007; Humphries et al., 2024; Parris & Beaudoin, 2004; Parris & Cornelius, 2004; Rachowicz & Vredenburg, 2004).

**TABLE 1:** Parameter values of two‐host susceptible‐infected compartmental models for *Litoria spenceri* and *Litoria lesueurii* at low, moderate, and high elevations, expressed as instantaneous daily rates.

Symbol	Description	*L. spenceri*			*L. lesueurii*			Refs.
		Low	Moderate	High	Low	Moderate	High	
S ^a^	Sex ratio	0.5	0.5	0.5	0.5	0.5	0.5	1
B_A1_ ^a^	Prob. female breeding in first adult year	0.5	0.5	0.5	0.5	0.5	0.5	2
B_A2_ ^a^	Prob. of female breeding in other adult years	1	1	1	1	1	1	2
*f*	Fecundity	0.144992	0.128279	0.108452	0.355074	0.370283	0.384531	3
$\delta_{L}$	Rate of aging from larva‐to‐juvenile	91.25^−1^	91.25^−1^	91.25^−1^	91.25^−1^	91.25^−1^	91.25^−1^	1
$\delta_{J}$	Rate of aging from juvenile‐to‐subadult	273.75^−1^	273.75^−1^	273.75^−1^	273.75^−1^	273.75^−1^	273.75^−1^	1
$\delta_{S},\delta_{A}$	Rate of aging through all post‐juvenile stages	365^−1^	365^−1^	365^−1^	365^−1^	365^−1^	365^−1^	1
$\beta_{L}$	Transmission rate in larvae	1 × 10^−5^	1 × 10^−5^	1 × 10^−5^	1 × 10^−5^	1 × 10^−5^	1 × 10^−5^	4
$\beta_{J}$	Transmission rate in juveniles	1 × 10^−6^	1 × 10^−6^	1 × 10^−6^	1 × 10^−6^	1 × 10^−6^	1 × 10^−6^	4
$\beta_{S}$	Transmission rate in subadults	1 × 10^−6^	1 × 10^−6^	1 × 10^−6^	1 × 10^−6^	1 × 10^−6^	1 × 10^−6^	4
$\beta_{A}$	Transmission rate in adults	1 × 10^−6^	1 × 10^−6^	1 × 10^−6^	1 × 10^−6^	1 × 10^−6^	1 × 10^−6^	4
$m_{L}$	Baseline mortality in larvae	0.012356	0.012356	0.012356	0.0081	0.0081	0.0081	1, 5
$m_{J}$	Baseline mortality in juveniles	0.012356	0.012356	0.012356	0.0081	0.0081	0.0081	1, 5
$m_{S1}$	Baseline mortality in 1st year subadults	0.001703	0.001359	0.001134	0.000962	0.000702	0.000622	5
$m_{S2}$	Baseline mortality in 2nd year subadults	0.001613	0.001292	0.001081	…	0.000642	0.000571	5
$m_{S3}$	Baseline mortality in 3rd year subadults	…	0.001013	0.000862	…	…	0.000350	5
$m_{S4}$	Baseline mortality in 4th year subadults	…	…	0.000862	…	…	…	5
$m_{A}$	Baseline mortality in adults	0.001214	0.001001	0.000855	0.000468	0.000375	0.000344	5
$\mu_{L}$	Added Bd mortality in larvae	0	0	0	0	0	0	4
$\mu_{J}$	Added Bd mortality in juveniles	0.000557	0.000814	0.001013	0.002364	0.002861	0.003046	5
$\mu_{S1}$	Added Bd mortality in 1st year subadults	0.000557	0.000814	0.001013	0.002364	0.002861	0.003046	5
$\mu_{S2}$	Added Bd mortality in 2nd year subadults	0.000581	0.000825	0.001013	…	0.002964	0.003139	5
$\mu_{S3}$	Added Bd mortality in 3rd year subadults	…	0.00087	0.001017	…	…	0.003587	5
$\mu_{S4}$	Added Bd mortality in 4th year subadults	…	…	0.001017	…	…	…	5
$\mu_{A}$	Added Bd mortality in adults	0.000684	0.00087	0.001017	0.003226	0.003488	0.003597	5
$\gamma_{L}$	Rate of infection loss in larvae	0	0	0	0	0	0	4
$\gamma_{J}$	Rate of infection loss in juveniles	0.008558	0.008558	0.008558	0.008153	0.008153	0.008153	5
$\gamma_{S}$	Rate of infection loss in subadults	0.008558	0.008558	0.008558	0.008153	0.008153	0.008153	5
$\gamma_{A}$	Rate of infection loss in adults	0.008558	0.008558	0.008558	0.008153	0.008153	0.008153	5
$\lambda_{L}$	Zoospore shedding rate in larvae	0.05	0.05	0.05	0.05	0.05	0.05	4
$\lambda_{J}$	Zoospore shedding rate in juveniles	0.05	0.05	0.05	0.05	0.05	0.05	4
$\lambda_{S}$	Zoospore shedding rate in subadults	0.05	0.05	0.05	0.05	0.05	0.05	4
$\lambda_{A}$	Zoospore shedding rate in adults	0.05	0.05	0.05	0.05	0.05	0.05	4
$\gamma_{z}$	Off‐host–zoospore decay rate	0.5	0.5	0.5	0.5	0.5	0.5	4, 6, 7

*Note*: References are: 1, Gillespie (2010); 2, Gillespie (2011a); 3, Estimated numerically by matching disease‐free growth rates from West et al. (2020); see *Methods*; 4, Theoretical value informed by plausibility of model output and/or organism life history, see *Methods*; 5, West et al. (2020); 6, Piotrowski et al. (2004); 7, Johnson and Speare (2003).

^a^ Indicates parameters that are scalar values rather than instantaneous daily rates.

#### Birth rates

As the conversion of finite birth rates to instantaneous birth rates requires strict assumptions even in populations with relatively simple life histories (Caswell, 1972), we calculated daily instantaneous fecundity numerically rather than analytically. With the compartmental models described thus far (but in the absence of Bd, carrying capacities, and seasonality in births), we used the bisection method (Burden & Faires, 1985) to iteratively converge on a per capita instantaneous fecundity which could replicate Bd‐free finite population growth rates intrinsic to the population matrix models described by West et al. (2020). Instantaneous fecundity values for each species/elevation combination were multiplied by sex ratio and probability of female breeding to calculate $\alpha_{k}$—the instantaneous daily birth rate for a given adult life stage *k*. To further account for seasonality in larval hatching, which may be an important consideration in infection rates of adults (Briggs et al., 2010), we multiplied instantaneous daily births by a truncated sinusoidal seasonal forcing function in both species which forces all annual births to occur from December through April, reaching a peak rate in late February (Appendix S1: Figure S1). The seasonally forced instantaneous daily hatching rate from adult stages therefore can be described by the following equation where k represents either adult stage, C represents the seasonal forcing coefficient, f represents the instantaneous daily fecundity, S represents the sex ratio, and B represents the probability of breeding:

(6) $$\begin{array}{c} \alpha_{k}\left(t\right)=C\left(t\right)\times f\times S\times B_{k} \end{array}.$$

We additionally incorporated a host carrying capacity into our models to prevent unrealistic exponential growth. Introducing a carrying capacity (*K*) as a function of adult density creates unrealistic Bd‐free behavior in this stage‐structured model, causing hatching rates to decline and annual larval cohort size to decrease as adult density approaches *K*. In real systems, limited breeding habitat (i.e., finite sites suitable for calling and oviposition), larval resource competition, and predation of larvae, should cause the larval cohort size to saturate as adult density increases (rather than decline). For this reason, we introduced a carrying capacity as a function of larval density rather than adult density for both species. Given that little is known regarding interspecific competition among larvae in this system, we modeled *K* separately for each species. We also assumed *K* to be equal across species and elevations.

#### Transmission, shedding, and zoospore decay

As *Z* in our density‐dependent model can represent any arbitrary unit of zoospore density, the values of transmission rate $\beta_{k}$ and shedding rate $\lambda_{k}$ were only influential (1) relative to one another and (2) relative to each life stage. We therefore optimized these parameters iteratively such that prevalence of infection across baseline quasi‐equilibrated model runs remained at intermediate levels similar to those observed in the wild (M. West, personal observations). Because larvae reside in water where zoospores exhibit active chemotaxis toward hosts (Moss et al., 2008), we set the average larval transmission rate $\beta_{L}$ as 10× greater than in post‐metamorphic stages. While larval transmission rate is likely higher than post‐metamorphic animals, relative shedding rates across life stages cannot be reasonably inferred from available data. Larval Bd infection intensities are higher than post‐metamorphic stages in some species but lower in others (Briggs et al., 2010; Gervasi et al., 2013; Hollanders et al., 2024), and post‐metamorphic infection intensities are not consistently associated with body size in the same manner across taxa (Searle et al., 2011). We therefore drew shedding rates separately for each life stage, but from the same probability distribution (see *Latin hypercube generation of parameter sets*).

Given the absence of experimental infection data to justify differences in within‐host Bd dynamics of each species, (1) we used a classically structured susceptible‐infected model rather than a more complex approach such as integral projection modeling (Wilber et al., 2016), and (2) we made no assumptions regarding the relative transmission and shedding rates of *L. spenceri* and *L. lesueurii* in our models, drawing parameter values separately for each species but from the same transmission and shedding rate probability distributions across Latin hypercube samples. Zoospore lifespan was set to an average of two days, which is longer than the time over which zoospores can maintain chemotaxis toward hosts (Piotrowski et al., 2004) but much shorter than the potential lifespan of sessile zoospores (Johnson & Speare, 2003). Although sessile zoospores often maintain infective capacity for weeks, a relatively fast decay rate was selected to better account for the downstream exit of zoospores in lotic host systems. A minimum ambient density $Z_{\min}$ = 1 zoospore unit was set to ensure realistic invasion and persistence of Bd as an endemic pathogen.

#### Latin hypercube generation of parameter sets

To evaluate system dynamics across uncertainty in model parameters, we used Latin hypercube sampling to generate *N* = 10,000 plausible parameter sets for each elevation using the R package “lhs” (Carnell & Carnell, 2016). With two exceptions, all individual parameter distributions were assumed to follow a normal distribution with mean value μ taken from literature values (Table 1) and a SD of μ/5, as such:

(7) $$\begin{array}{c} \text{parameter}\;˜\;\text{Normal}\left(\mu ,\frac{\mu }{5}\right). \end{array}$$

Meanwhile, shedding rates $\lambda_{k}$ and transmission rates $\beta_{k}$ were sampled from a more conservative uniform distribution covering two orders of magnitude, as shown below:

(8) $$\begin{array}{c} \lambda_{k}˜\text{uniform}\left(0.1\lambda_{k},10\lambda_{k}\right) \end{array},$$

(9) $$\begin{array}{c} \beta_{k}˜\text{uniform}\left(0.1\beta_{k},10\beta_{k}\right) \end{array}.$$

The only parameters maintained as constant values across all simulations were sex ratio, probability of breeding, stage transition rates, and minimum ambient density of zoospores.

### Model execution

#### Quasi‐equilibration and realism filtering of parameter sets

To bypass transient dynamics from arbitrarily assigned initial compartment densities, models for all Latin hypercube sample parameter sets were run individually to a quasi‐equilibrated state before simulations of management interventions. Quasi‐equilibrium was satisfied when both species exhibited an annual change in adult population size ≤1% for 20 consecutive years. To account for the special case where one species moving indefinitely toward extinction could not meet this criterion (i.e., population densities <1), quasi‐equilibrium was also assumed when the adult ratio between species surpassed 1:100. Quasi‐equilibrated parameter sets at each elevation were discarded for lack of realism if (1) the final ratio of *L. lesueurii* adults to *L. spenceri* adults was greater than 50, or (2) the adult prevalence of infection fell outside a 5%–70% range in either species—evaluated in both winter (July 2) and summer (January 1) in the year of quasi‐equilibrium.

#### Simulation of management interventions

To evaluate the relative impacts of (1) *L. lesueurii* removal, (2) non‐native trout removal, and (3) *L. spenceri* captive breeding and release on *L. spenceri* population outcomes, we simulated three 20‐year management scenarios for each elevation. Management scenarios were simulated at 10% increments of intervention, using all quasi‐equilibrated parameter sets which passed realism criteria. At each increment, the median adult densities of both species in the 20th year were used to evaluate management outcomes across realistic parameter sets. The *L. lesueurii* removal scenario was simulated by increasing *L. lesueurii* post‐metamorphic mortality at 10% increments from its baseline (0% intervention) until matching the higher mortality exhibited by *L. spenceri* (100%). While the true trout‐free baseline mortality of *L. spenceri* larvae cannot be estimated from field data, this value was assumed to approximate the larval baseline mortality of *L. lesueurii*—a sympatric species which does not experience significant trout predation (Gillespie, 2001). Additionally, because the larval and juvenile baseline mortality could not be parameterized separately, the impact of reduced trout predation had to be evaluated in tandem over both life stages. The reduced trout predation scenario was therefore implemented by decreasing the baseline mortality of larval and juvenile *L. spenceri* from its baseline (0% intervention) until matching that of *L. lesueurii* (100% intervention). Captive breeding interventions, meanwhile, simulated the effects of releasing newly hatched *L. spenceri* larvae into streams. The captive breeding management scenario was therefore implemented by raising the innate fecundity of *L. spenceri* (0% intervention) until matching the higher innate fecundity of *L. lesueurii* (100% intervention).

We additionally evaluated the interactive impact of trout removal and captive breeding/release on pathogen‐mediated competition between the two species using a contour plot. Using the median parameter values across all the quasi‐equilibrated parameter sets which fulfilled realism criteria, we ran a 100 × 100 grid of 10,000 incremented management interventions to their new quasi‐equilibrium states. Both baseline larval/juvenile *L. spenceri* mortality and *L. spenceri* fecundity were varied at 1% increments from the innate parameter values for *L. spenceri* (0% intervention) until matching the innate parameter values for *L. lesueurii* (100%) intervention.

#### Sensitivity analysis

At each elevation, partial rank correlation coefficients (PRCCs) were calculated using the R package “epiR” (Stevenson et al., 2023) to determine the impact of model parameters on six outcomes of quasi‐equilibrated systems: (1) adult density of *L. spenceri*, (2) adult density of *L. lesueurii*, (3) competitive success of *L. spenceri*—defined as the proportion of the total adult frog community which is *L. spenceri*, (4) prevalence of infection in *L. spenceri* adults, (5) prevalence of infection in *L. lesueurii* adults, and (6) density of Bd zoospores in the environment. Negative PRCCs represent the life history and infection parameters that most negatively influence system outcomes, while positive PRCCs indicate positive associations with system outcomes. Simplified composite PRCCs were calculated by averaging PRCCs across all life stage variants and all elevations for a given parameter.

## RESULTS

### Model diagnostics

Of the 10,000 Latin hypercube sample parameter sets used for each elevation, 4501, 7061, and 8252 quasi‐equilibrated models passed realism criteria for high, moderate, and low elevations, respectively. Both quasi‐equilibrated life stage densities and the concentration of Bd zoospores were greater at lower elevations (Appendix S1: Figure S2). The only exception to this trend was the subadult stage, which is expected given that time spent in the subadult life stage is positively correlated with elevation. Concomitant with a surge in larval density following the breeding season, adult prevalence of infection for both species was higher on average in winter than in summer, with mean prevalence across species/elevations ranging from 17.9% to 23.8% in summer and 31.4% to 42.2% in winter (Appendix S1: Figure S3). The median ratio of *L. lesueurii* adults to *L. spenceri* adults at quasi‐equilibrium was 8.24‐to‐1 for high elevations, 6.26‐to‐1 for moderate elevations, and 5.44‐to‐1 for low elevations (Appendix S1: Figure S4).

### Management scenarios

**FIGURE 2:**
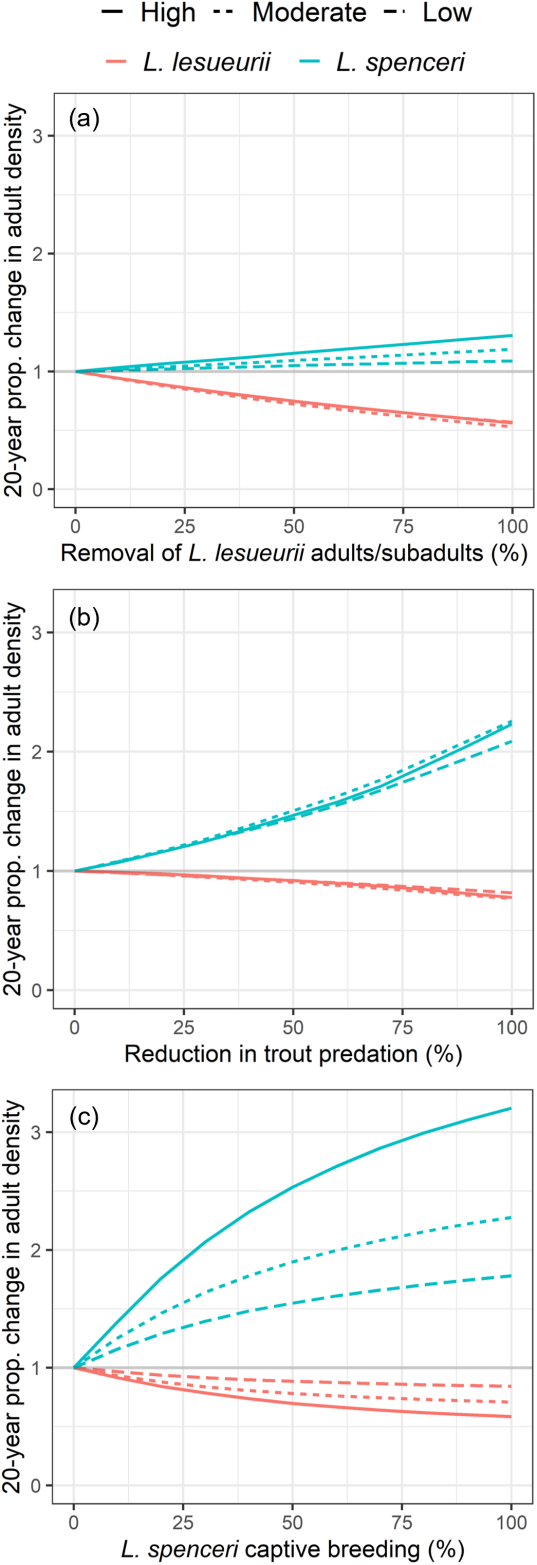
Proportional change in median adult density of *Litoria spenceri* and *Litoria lesueurii* at high, moderate, and low elevations after 20‐year simulations of three alternative management interventions: (a) removal of *L. lesueurii* frogs, simulated by increasing post‐metamorphic *L. lesueurii* mortality from its baseline value (0% intervention) to that of *L. spenceri* (100% intervention), (b) reduced predation by non‐native trout, simulated by reducing *L. spenceri* larval and juvenile mortality from their baseline values (0% intervention) to those of *L. lesueurii* (100% intervention), (c) captive breeding and release of *L. spenceri* tadpoles, simulated by increasing *L. spenceri* fecundity from its baseline value (0% intervention) to that of *L. lesueurii* (100% intervention). At each 10% increment from 0% to 100% intervention, 20‐year management simulations were run for all quasi‐equilibrated parameter sets which passed realism criteria.

In all three 20‐year management scenarios, higher degrees of intervention increased *L. spenceri* adult density and decreased *L. lesueurii* adult density (Figure 2). While efficacy of *L. lesueurii* removal and captive breeding improved clearly with increasing elevation (Figure 2a,c), only marginal differences in efficacy were observed across elevations for trout removal (Figure 2b). Removal/exclusion of *L. lesueurii* adults consistently reduced the density of Bd zoospores in the environment (Appendix S1: Figure S6); however, it was also the least beneficial intervention for *L. spenceri* and the most detrimental to *L. lesueurii* on average across elevations (Figure 2a). Alternatively, captive breeding/release and trout removal consistently elevated the density of Bd zoospores in the environment (Appendix S1: Figures S7 and S8) whilst simultaneously increasing *L. spenceri* density and decreasing *L. lesueurii* density (Figure 2b,c). Twenty years of either captive breeding or trout removal at 100% intervention resulted in adult *L. spenceri* populations ranging from 1.8 to 3.2 times larger than scenarios without intervention (Figure 2b,c). In high elevations at 100% intervention, *L. spenceri* captive breeding was almost twice as effective for *L. spenceri* recovery as reducing trout predation; however, the reverse was true at low elevations, where reducing trout predation benefitted *L. spenceri* slightly more than captive breeding (Figure 2b,c).

**FIGURE 3:**
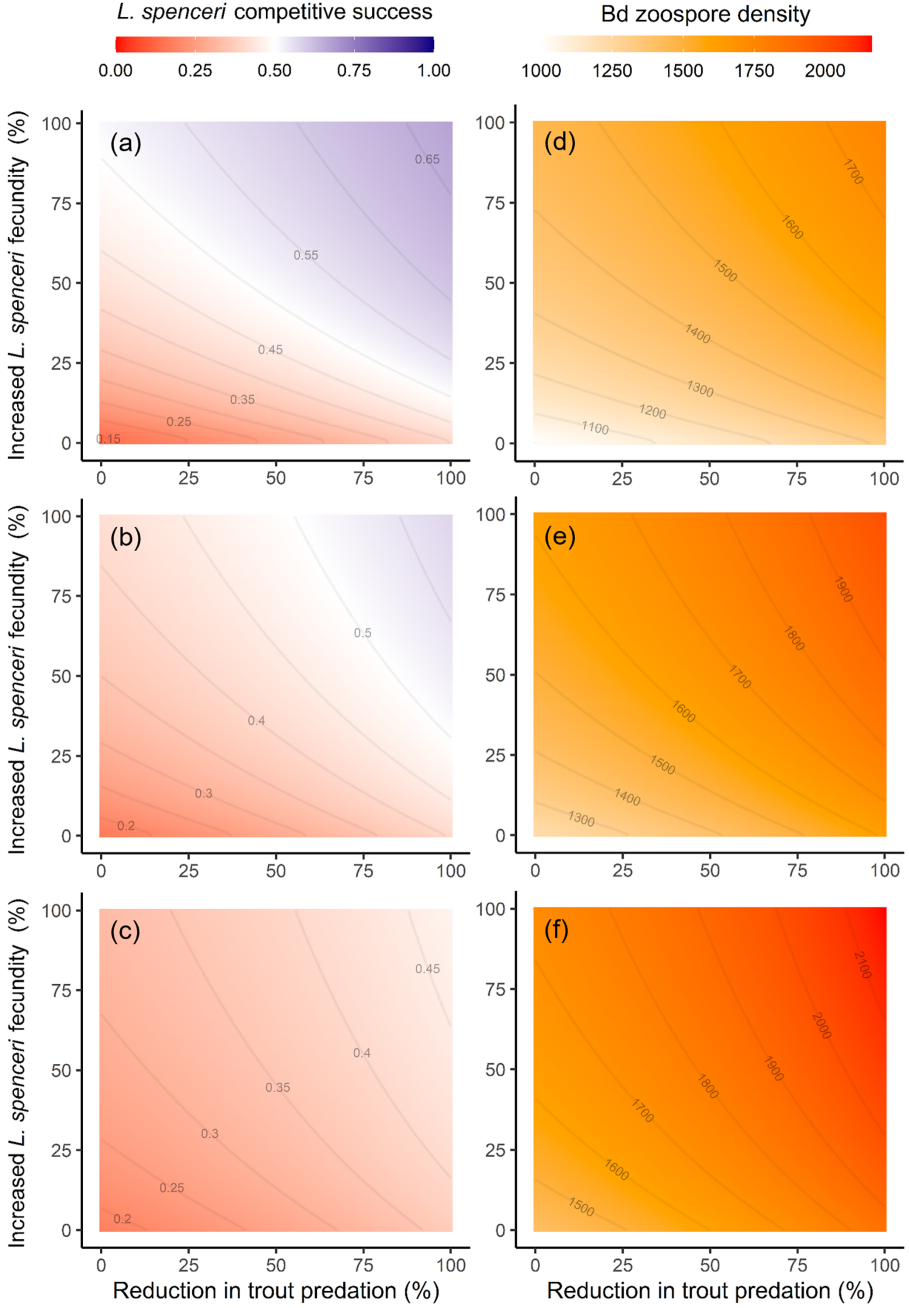
Contour plots displaying quasi‐equilibrated system outcomes across varying degrees of intervention to reduce trout predation and increase *Litoria spenceri* fecundity across (a, d) high, (b, e) moderate, and (c, f) low elevations. Intervention to bolster *L. spenceri* fecundity was scaled from the innate fecundity of *L. spenceri* (0% intervention) until matching the innate fecundity of *Litoria lesueurii* (100% intervention) for each elevation. (a–c) *Litoria spenceri* “competitive success” is the proportion of the total community adult density that is comprised by *L. spenceri* [Adult_*spencer*i_/(Adult_*spenceri*_ + Adult_*lesueurii*_)]. Red values indicate that *L. spenceri* adult density is lower than that of *L. lesueurii*, while blue values indicate that *L. spenceri* adult density is higher than *L. lesueurii*. (d–f) Density of Bd zoospores in the zoospore pool across different levels of intervention, where red values represent higher density.

Combined trout removal and *L. spenceri* captive breeding/release consistently improved the quasi‐equilibrium competitive success of *L. spenceri* relative to *L. lesueurii*, and this effect became more pronounced with increasing elevation—in accordance with the fact that captive breeding efficacy was positively associated with elevation (Figure 3a–c). *L. spenceri* became the most abundant species in both moderate‐ and high‐elevation simulations where both trout removal and captive breeding were employed together at high degrees of intervention (Figure 3a,b). Improved *L. spenceri* competitive success from combined captive breeding and trout removal was consistently associated with an increase in the density of Bd zoospores in the environment (Figure 3d–f).

### Sensitivity analysis

**FIGURE 4:**
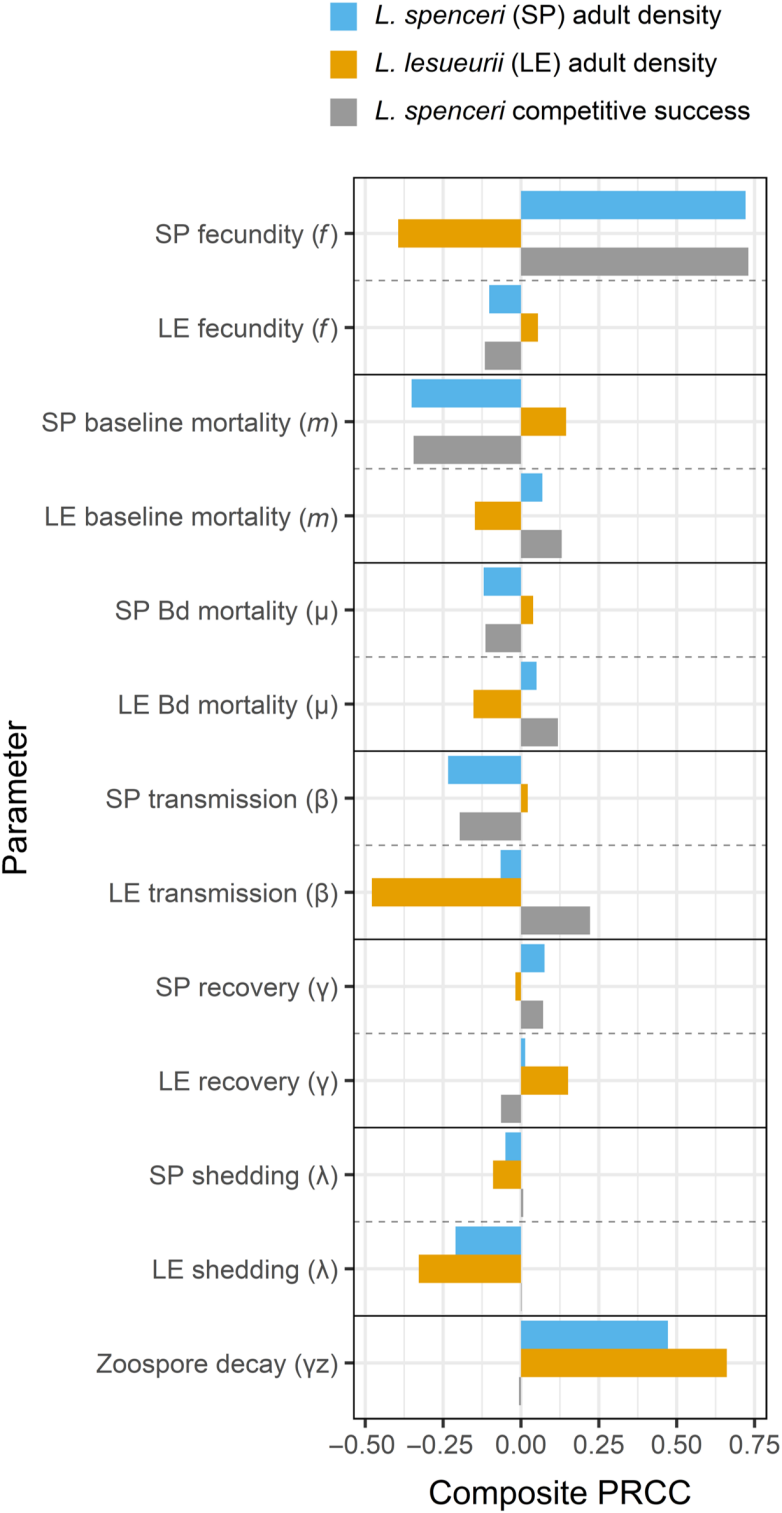
Composite partial rank correlation coefficients (PRCCs) for each parameter type in the model according to three outcomes: adult density of *Litoria spenceri* (SP), adult density of *Litoria lesueurii* (LE), and competitive success of *L. spenceri*, defined as the proportion of the total community adult density that is comprised by *L. spenceri* [Adult_*spenceri*_/(Adult_*spenceri*_ + Adult_*lesueurii*_)]. Negative values indicate that the parameter has a negative association with a given outcome across parameter space, while positive values indicate a positive association. Composite PRCCs are averages of PRCCs for all life stage variants of a given parameter across all elevations. The full‐sensitivity analysis is displayed in Appendix S1: Figure S9.

According to partial rank correlation coefficients (PRCCs), parameters related to recruitment showed the strongest relationship with *L. spenceri* adult density, while parameters related to infection showed the strongest relationship with *L. lesueurii* adult density (Figure 4). Composite PRCCs of both adult density and competitive success of *L. spenceri* were most positively associated with *L. spenceri* fecundity and most negatively associated with *L. spenceri* baseline mortality (Figure 4). Meanwhile, *L. spenceri* infection parameters showed weak PRCCs except in the case of second‐year adults—the only stage with full reproductive capacity (Appendix S1: Figure S9). Alternatively, *L. lesueurii* density primarily showed a strong positive association with zoospore decay rate and negative associations with *L. lesueurii* transmission/shedding rates and *L. spenceri* fecundity (Figure 4). The negative association of *L. lesueurii* transmission with its respective adult density was over two times larger than that of *L. spenceri* (Figure 4). While parameters directly influencing zoospore density in the environment (shedding rate, zoospore decay) were impactful individually on *L. spenceri* and *L. lesueurii* outcomes, these parameters had almost no effect on *L. spenceri* competitive success (Figure 4). For each species, prevalence of infection was almost entirely mediated by transmission rate of the adult stages, shedding rate of *L. lesueurii*, and zoospore decay rate (Appendix S1: Figure S9). Density of Bd zoospores in the environment was similarly most influenced by *L. lesueurii* shedding and zoospore decay rate (Appendix S1: Figure S9).

## DISCUSSION

Our models demonstrate a context where relative rates of recruitment between species, rather than infection parameters, drive outcomes of pathogen‐mediated competition in an amphibian chytridiomycosis system. Despite the non‐declining *L. lesueurii* showing higher Bd‐associated mortality and infection rates than the critically endangered *L. spenceri* in the wild (West, 2015), it has been suggested that *L. lesueurii* acts as a Bd reservoir or amplification host which exacerbates *L. spenceri* declines (West et al., 2020, 2024). Recent dynamic occupancy modeling provided strong observational evidence in support of this hypothesis, showing that combined presence of Bd and *L. lesueurii* increases risk of *L. spenceri* extirpation (West et al., 2024). While previous fixed‐rate population matrix models suggest that substantially higher rates of recruitment may explain the success of *L. lesueurii* populations relative to the critically endangered *L. spenceri*, the capacity for *L. lesueurii* to act as a reservoir has not been explored mechanistically. Here, using a susceptible‐infected modeling framework that explicitly accounts for cross‐species infection dynamics, we demonstrate that demographic characteristics (rather than susceptibility to infection and disease) permit *L. lesueurii* to act as a Bd‐amplifying host which exacerbates *L. spenceri* declines. This outcome is predominantly driven by species differences in (1) reproductive rate, (2) predation of early life stages by non‐native trout, and (3) the impact of elevation‐based life history on pathogen amplification. Encouragingly for *L. spenceri* conservation, the degree to which *L. lesueurii* acts as an amplifying host is dependent on ecological context such that simulated management interventions (i.e., captive breeding and release, trout removal) successfully mitigated and even reversed the outcome of pathogen‐mediated competition between these species. This system underscores how the interactive effects of disease, predation, and demography on amphibian conservation should be considered explicitly in the wider context of the amphibian community.

### Relative recruitment drives baseline competitive outcomes

While many amphibian hosts are competent Bd reservoirs and/or amplifying hosts due to their physiological tolerance of high zoospore loads (Adams et al., 2017; Brannelly et al., 2017; Reeder et al., 2012), multistate mark‐recapture modeling of wild populations shows that the non‐declining *L. lesueurii* paradoxically is more susceptible to infection, disease, and subsequent mortality in situ than the sympatric species it is thought to negatively impact (West, 2015). Increased susceptibility of *L. lesueurii* to disease was apparent in the sensitivity analysis of our baseline quasi‐equilibrated models, as the density of adult *L. lesueurii* was almost twice as sensitive to increases in its respective transmission rate than *L. spenceri*. Furthermore, parameters which indiscriminately increase the force of infection in the environment (i.e., zoospore lifespan and shedding rates) more negatively impacted *L. lesueurii* density than *L. spenceri*.

Since *L. spenceri* is therefore more competitive based on infection parameters alone, the clear dominance of *L. lesueurii* under baseline conditions, and its role as a Bd‐amplifier, is apparently driven entirely by its high relative rate of recruitment. This result is consistent with life history theory, which predicts that fast‐living species (i.e., faster generation time and higher fecundity) experience less population depression due to infectious disease than slow‐living species when both host and parasite fundamental recruitment numbers (*R*_0_ and *R*_0,host_) are constant (Valenzuela‐Sánchez et al., 2021). As the faster‐living species in our system, the clutch size of *L. lesueurii* ranges from 2.2 to 2.9 times higher than *L. spenceri* across elevations, and adults tend to mature a year earlier than *L. spenceri* in the wild (Gillespie, 2010, 2011a; G. Gillespie, unpublished data). Furthermore, non‐native trout selectively predate *L. spenceri* tadpoles, increasing the *L. lesueurii* rate of juvenile recruitment relative to its competitor (Gillespie, 2001). These higher vital rates allowed *L. lesueurii* to reach a median adult density between five and eight times higher than that of *L. spenceri* across our baseline quasi‐equilibrated models despite having higher susceptibility to disease. Given the community dominance of *L. lesueurii* under baseline conditions, *L. spenceri* outcomes accordingly were strongly impacted by *L. lesueurii* zoospore shedding rate but showed negligible sensitivity to shedding rate of its own life stages. Previous studies have identified high juvenile recruitment as a protective mechanism against Bd‐associated declines (Louca et al., 2014; Muths et al., 2011; Scheele et al., 2015; Valenzuela‐Sánchez et al., 2022); however, our results further illustrate that this carries a negative competitive consequence for other susceptible species in the amphibian community. By using recruitment as a buffer against disease‐associated mortality, *L. lesueurii* acts as an amplifying host which competently suppresses *L. spenceri* population growth despite having greater susceptibility to disease.

While the non‐threatened *L. lesueurii* amplifies the force of infection due to its high relative density, the status of *L. lesueurii* as a true reservoir host is contextually ambiguous in our model framework. A reservoir must permanently maintain and facilitate transmission of infection to a target species which cannot itself maintain the pathogen (Haydon et al., 2002; Roberts & Heesterbeek, 2020). While a high prevalence of infection and recruitment‐based buffering capacity against Bd mortality makes *L. lesueurii* a strong candidate to act as a reservoir, such traits are rarely sufficient to demonstrate reservoir potential without additional ecological context (Wilber, DeMarchi, et al., 2022). For example, although *Crinia signifera* acts as reservoir host driving declines of the critically endangered *Pseudophryne pengilleyi* (Brannelly et al., 2017; Scheele, Hunter, et al., 2017), it demonstrates poor reservoir competence in other amphibian communities and ecological contexts (Crawford‐Ash & Rowley, 2021). Specifically in our management simulations, the complete removal of non‐native trout caused simultaneous increases in both *L. spenceri* competitive success and Bd zoospore density, suggesting that *L. spenceri* may independently act as a competent maintenance host in sites where larval predation is reduced. Our models also do not account for additional species that may co‐occur with *L. spenceri* (e.g., *L. paraewingii*, *L. nudidigitus*, and *C. signifera*), which may function in concert as a maintenance community even in cases where no species individually can maintain the pathogen (Roberts & Heesterbeek, 2020). For these reasons, we argue that description of *L. lesueurii* as an amplification host, rather than as a reservoir, is more contextually robust in understanding its negative impact on *L. spenceri* conservation.

### Recruitment‐based interventions modify pathogen‐mediated competition

While removal or exclusion of reservoir/amplification hosts has been posited as a management strategy to protect vulnerable species (Skerratt et al., 2016), benefits of this approach were modest in our simulations compared with recruitment‐based interventions. Removal of post‐metamorphic *L. lesueurii* facilitated *L. spenceri* population growth in our simulations by reducing the overall density of zoospores in the environment; however, massive losses in *L. lesueurii* density provided only moderate 20‐year growth of the *L. spenceri* population. Given these mild benefits as well as the difficulty of excluding reservoir hosts in natural populations (Klop‐Toker et al., 2021), direct removal of *L. lesueurii* has limited practicality as a management intervention for *L. spenceri* recovery. It should be noted, however, that this conclusion may not be widely generalizable to other reservoir systems given the unusual role of *L. lesueurii* as a pathogen amplifier which is also highly susceptible to disease. Specifically, each infected *L. lesueurii* will have a limited contribution to the force of infection before disease proves fatal, whereas physiologically tolerant hosts such as *C. signifera* and *L. catesbeiana* can continue to shed zoospores indefinitely while infection persists (Brannelly et al., 2017; Daszak et al., 2004; Hanselmann et al., 2004; Miaud et al., 2016; Scheele, Hunter, et al., 2017; Schloegel et al., 2010). For this reason, removing tolerant amplifiers would likely reduce the cumulative force of infection more substantially per individual than the removal of susceptible amplifiers like *L. lesueurii*. This claim is consistent with observational evidence, as the prevalence of infection in Bd‐vulnerable amphibians is often positively associated with the presence of co‐occurring tolerant species (Borzée et al., 2017; Fernandez‐Beaskoetxea et al., 2016; Hossack, Oja, et al., 2023; Kärvemo et al., 2019; Peterson & McKenzie, 2014; Scheele, Hunter, et al., 2017). While few removal programs of Bd‐tolerant hosts have been completed, a near‐total eradication of invasive *L. catesbeiana* from amphibian communities in Arizona resulted in coextirpation of both Bd and ranavirus across all formerly infected experimental sites (Hossack, Hall, et al., 2023). This provides strong evidence that interventions to remove or exclude amplification/reservoir species can be effective in the appropriate ecological context.

As opposed to the limited benefits of removing an amplifying species, our management simulations demonstrated that increasing rates of recruitment can substantially change outcomes of pathogen‐mediated competition in an amphibian community. Captive breeding and trout removal consistently resulted in strong recoveries of the critically endangered *L. spenceri* across elevations, and these interventions even allowed *L. spenceri* to become the most abundant species in the adult community across at moderate and high elevations when employed concurrently at 100% intervention. Furthermore, beyond merely slowing Bd‐associated mortality (Louca et al., 2014; Muths et al., 2011; Scheele et al., 2015), our simulations demonstrate that the pathogen pressure applied between competing species is heavily influenced by recruitment‐based interventions. Even though (1) neither scenario changed *L. lesueurii* parameters and (2) direct species competition was nonexistent in our model framework, *L. lesueurii* exhibited a clear decline in population density and competitive success in response to interventions that bolster *L. spenceri* recruitment. Additionally, in opposition to *L. lesueurii* removal as an intervention, both captive breeding and trout removal caused *higher* Bd density in the environment. In aggregate, this indicates that increased *L. spenceri* recruitment enhances the Bd‐mediated competitive pressure that it can apply onto sympatric species and, in so doing, further mitigates the threat of *L. lesueurii* as an amplifying host—a positive feedback loop. While this qualitative trend is clear in a theoretical context, the strength of this relationship in the wild may be further mediated by poorly studied factors which were not included in our models, chiefly direct interspecific competition, impacts of disease morbidity on vital rates, and the potential for differential transmission and zoospore shedding rates across species.

Lastly, when comparing management simulations across our three model structures, it is clear that elevation may potentially modulate pathogen‐mediated competition. Most notably, management efficacy of *L. spenceri* captive breeding and *L. lesueurii* removal consistently increased with elevation. Given that our models did not include elevation‐dependent environmental variables (i.e., temperature, humidity) which are known to alter Bd growth and infection dynamics (Greenberg et al., 2017; Murray et al., 2011; Stevenson et al., 2013), the increased efficacy of interventions at high elevations can be entirely attributed to associated changes in life history. Specifically, reproductive rates of both species (i.e., fecundity and generation time) are faster at lower elevations, which concomitantly increases total host density, zoospore density, and prevalence of infection. This may bolster the absolute impact of *L. lesueurii* as an amplifying host and constrain the leverage of low elevation management interventions. While trout removal displayed only marginal differences in efficacy across elevations, comparison of our results with that of fixed‐rate matrix models in West et al. (2020) further supports the presence of an elevation effect on pathogen‐mediated competition. Fixed‐rate matrix models (without dynamic cross‐species transmission) suggested that the benefit of trout removal increases at *lower* elevations (West et al., 2020); however, our model framework (with dynamic cross‐species transmission) entirely mitigates this elevation effect in management outcomes. This is consistent with a heightened impact of *L. lesueurii* as an amplifying host at low elevations, ultimately undercutting the benefits of trout removal. Such model framework comparisons highlight the importance of considering cross‐species infection dynamics when examining a multi‐host pathogen system.

While elevation impacts pathogen‐mediated competition in our theoretical model framework, elevation‐based differences in simulated management outcomes should be extrapolated cautiously to real systems. Most notably, the high sensitivity of our baseline quasi‐equilibrated models to zoospore decay rate implies that elevation‐based differences in Bd viability could strongly influence community dynamics. Given that Bd growth is diminished above 25°C (Piotrowski et al., 2004), summertime infection prevalence tends to dip more sharply at warmer lower elevations than at cooler higher elevations (Kriger & Hero, 2008; Sapsford et al., 2013). Such seasonal fluctuations in environmental conditions often have greater impact on infection dynamics and disease outcomes than overall population density (Wilber, Knapp, et al., 2022). Accordingly, this may mitigate the summer impact of *L. lesueurii* as an amplifying host at low elevations despite its higher vital rates. Furthermore, although higher reproductive rates at low elevations lead to higher overall population densities in our results, this is not always true of real populations (M. West & G. Gillespie, personal observations). Other important factors unaccounted for in our modeling framework (e.g., habitat suitability and metapopulation dynamics) impact occupancy and relative abundance of these species across sites.

### Model limitations

While our model outcomes demonstrate key qualitative features of pathogen‐mediated competition in the *L. lesueurii*/*L. spenceri* system, the model structure and parameterization make substantial simplifying assumptions. Most notably, our modeling framework was agnostic to species differences in Bd transmission rate, shedding rate, initial infection burden, and within‐host Bd growth rate. Some recent Bd epidemiological models have explicitly accounted for these factors using laboratory infection data and/or integral projection modeling (Briggs et al., 2010; Wilber et al., 2016, 2017, 2020; Wilber, Ohmer, et al., 2022). However, as laboratory and field infection data were unavailable to inform such parameters in our study system, we opted instead for a simplified susceptible‐infected model where these infection parameters were drawn from the same probability distribution in each species.

Spatial distribution of hosts may also be an important consideration in this amphibian system. While *L. spenceri* is closely associated with stream banks (Gillespie & Hollis, 1996), *L. lesueurii* moves farther from streams than other sympatric *Litoria* spp. and frequently traverses non‐forested, disturbed habitat (Rowley & Alford, 2007a). Such increased movement in *L. lesueurii* could mitigate the impact of Bd on this species relative to our field‐based estimates from stream surveys, as individuals farther from areas of higher amphibian density (i.e., riparian zones) may have lower Bd contact and transmission rates. *L. lesueurii* also exhibits less contact with water outside of the breeding season, meaning their shedding contribution to the zoospore pool may lessen from January to August (Rowley & Alford, 2007a, 2007b). Also, we do not account for aggregation behavior which may deviate from the assumptions of density‐dependent transmission in our models. Post‐metamorphic *L. spenceri* are often observed basking together on sunny days, sometimes in direct physical contact, and adult *L. lesueurii* frequently aggregate on rocky bank areas (G. Gillespie & M. West, personal observations). There is additionally some evidence of *L. spenceri* aggregation in shared hibernacula (G. Gillespie, personal observations), similarly to as observed in the congeneric *Litoria pearsoniana* (McDonald & Davies, 1990). Behaviors which increase host‐to‐host contact may result in some degree of frequency‐dependent transmission, which can drive species extinctions in the absence of reservoir hosts or other threats (De Castro & Bolker, 2005). Notably, one high‐density population of *L. spenceri* underwent extinction during a Bd epidemic in the absence of both predatory trout and *L. lesueurii* (Gillespie et al., 2015). Such extinctions could potentially result from unidentified reservoirs of Bd, non‐density‐dependent transmission, stochastic effects, or other conservation threats which were not detected (De Castro & Bolker, 2005). Overall, while the impact of recruitment on pathogen‐mediated competition is clear in our results and important to consider in the generalized amphibian context, applied management strategies in the narrow context of the *L. lesueurii*/*L. spenceri* system should consider additional features and site‐specific idiosyncrasies which were not examined in our modeling framework.

## CONCLUSIONS

Differential species susceptibility to infection and disease is undoubtedly a key determinant of infection dynamics in amphibian communities; however, our simulation models demonstrate an instance where variation in recruitment between species is the driving force behind pathogen‐mediated competition. Even though the non‐declining species *L. lesueurii* is more susceptible to Bd than its competitor, higher fecundity, faster generation time, and evasion of predator‐mediated juvenile mortality allow *L. lesueurii* to reach high densities in the amphibian community. With its high recruitment rate buffering against Bd‐associated mortality, *L. lesueurii* acts as an amplifying host and drives a less Bd‐susceptible species toward extinction. Captive breeding and removal of introduced predators consistently mitigated and sometimes reversed competitive outcomes in our management simulations, demonstrating that recruitment‐based interventions can situationally act as powerful tools of conservation management for Bd‐affected species.

More generally, our results emphasize the need for dynamic epidemiological models when assessing strategies to manage disease threats in multispecies systems. While a focus on removal of sympatric reservoir hosts or assisted selection toward resistance/tolerance to infection may be more prudent solutions in some amphibian communities, our findings highlight that classic demographic interventions should not be discounted. A mechanistic synthesis of community infection dynamics, interactive threats, and demographic context is essential in designing management interventions which most effectively mitigate disease‐associated declines.

## AUTHOR CONTRIBUTIONS

Madelyn J. Mangan contributed to conceptualization, model design, coding, formal analysis, visualization, writing (original draft), and writing (review and editing). Laura F. Grogan contributed to conceptualization, model design, writing (review and editing), and project supervision. Hamish I. McCallum contributed to conceptualization, model design, writing (review and editing), and project supervision. Matt West contributed to conceptualization and writing (review and editing). Ben C. Scheele contributed to conceptualization and writing (review and editing). Graeme R. Gillespie contributed to writing (review and editing).

## CONFLICT OF INTEREST STATEMENT

The authors declare no conflicts of interest.

## Supporting information


Appendix S1.

## Data Availability

Data and code (Mangan, 2024) are available on Zenodo: https://doi.org/10.5281/zenodo.13855820. This dataset was adapted in part from Table 1 in West et al. (2020), available at https://doi.org/10.1016/j.biocon.2019.108247.
