# Supplementary material for: Differential recruitment drives pathogen‐mediated competition between species in an amphibian chytridiomycosis system
Source: Ecol Appl. 2025 Jan 17;35(1):e3085. doi: 10.1002/eap.3085 (PMC11751701; doi:10.1002/eap.3085)
Supplement: Supplementary file 1 — Appendix S1. [file EAP-35-e3085-s001.pdf]

APPENDIX S1

Ecological Applications - Article

Differential recruitment drives pathogen-mediated competition between species in an amphibian chytridiomycosis system

Madelyn J. Mangan, Hamish I. McCallum, Matt West, Ben C. Scheele,

Graeme R. Gillespie, Laura F. Grogan

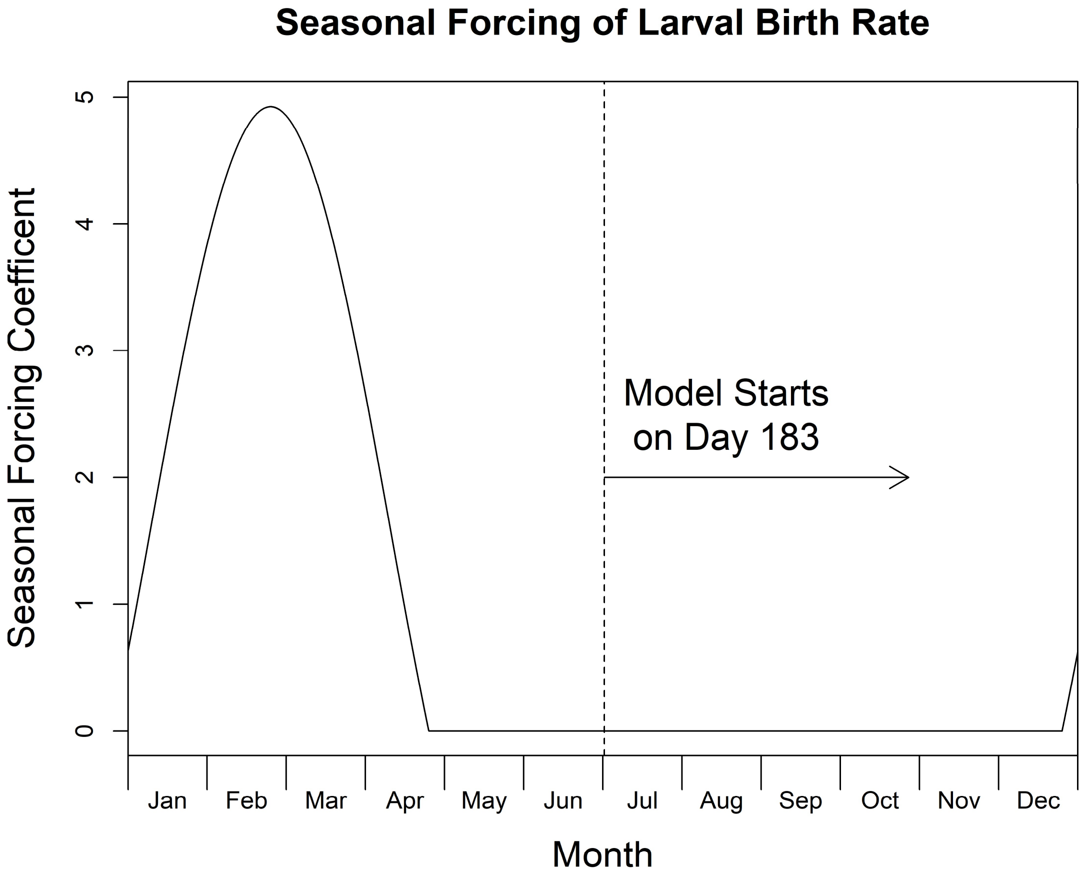

Figure S1. The seasonal forcing coefficient as a function of time for *L. spenceri* and *L. lesueurii* birth rate (hatching of larvae). Following a truncated sinusoidal function, annual hatching commences in late December and ends in late April. The model begins on the 183rd day of the year (July 2nd).

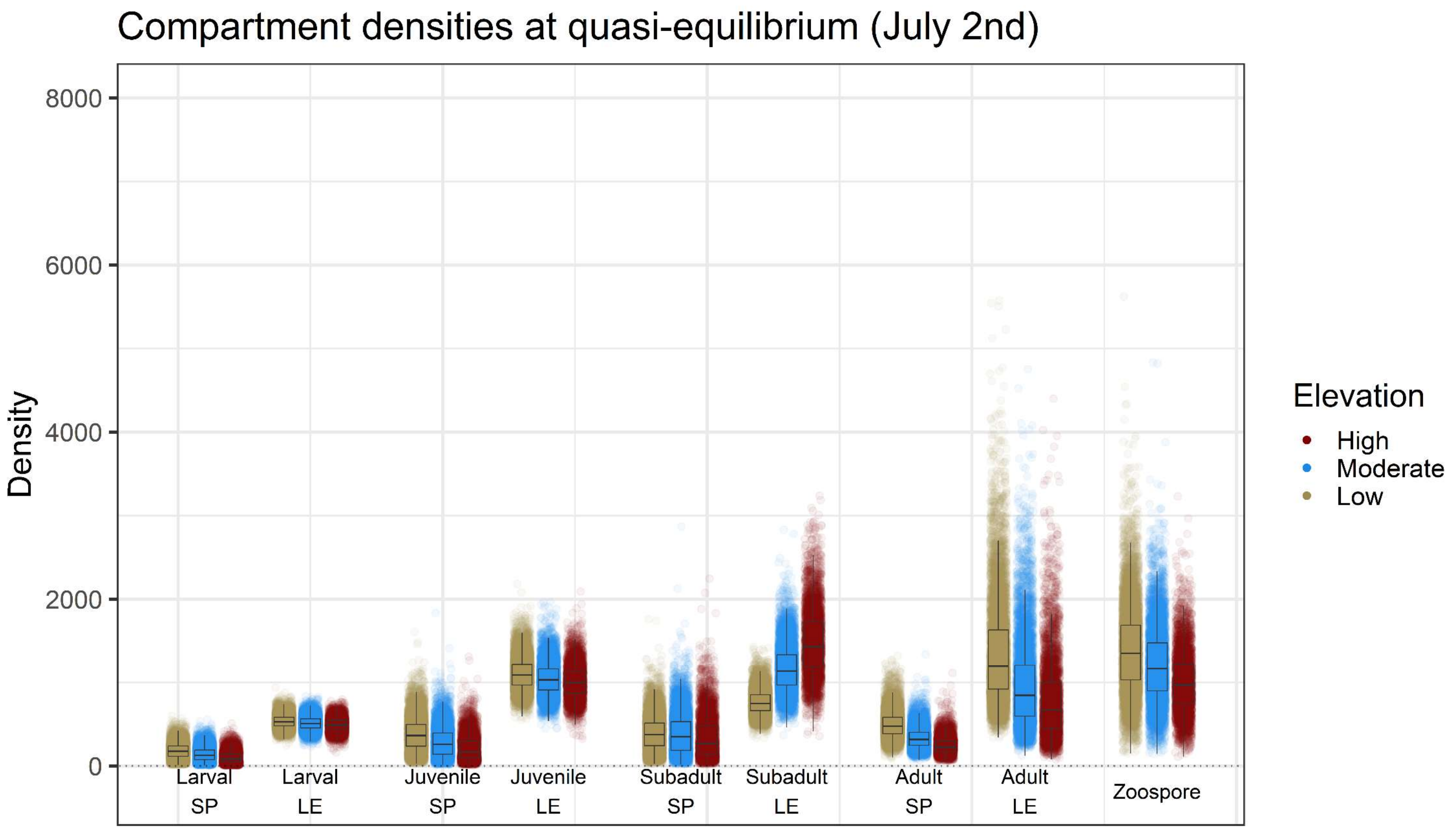

Figure S2. Compartment densities on July 2nd in the year of quasi-equilibrium for each species and life stage across low (gold), moderate (blue) and high (red) elevation baseline models. Each point represents the quasi-equilibrium outcomes for a single parameter set which met realism criteria. Boxplots display the median outcome and quartiles for each compartment. SP = *L. spenceri*; LE = *L. lesueurii*. (Note: as both subadult and adult stages are comprised of multiple compartments, these categories represent summed densities across all compartments of a given type. Higher elevation models have more subadult compartments than lower elevation models.)

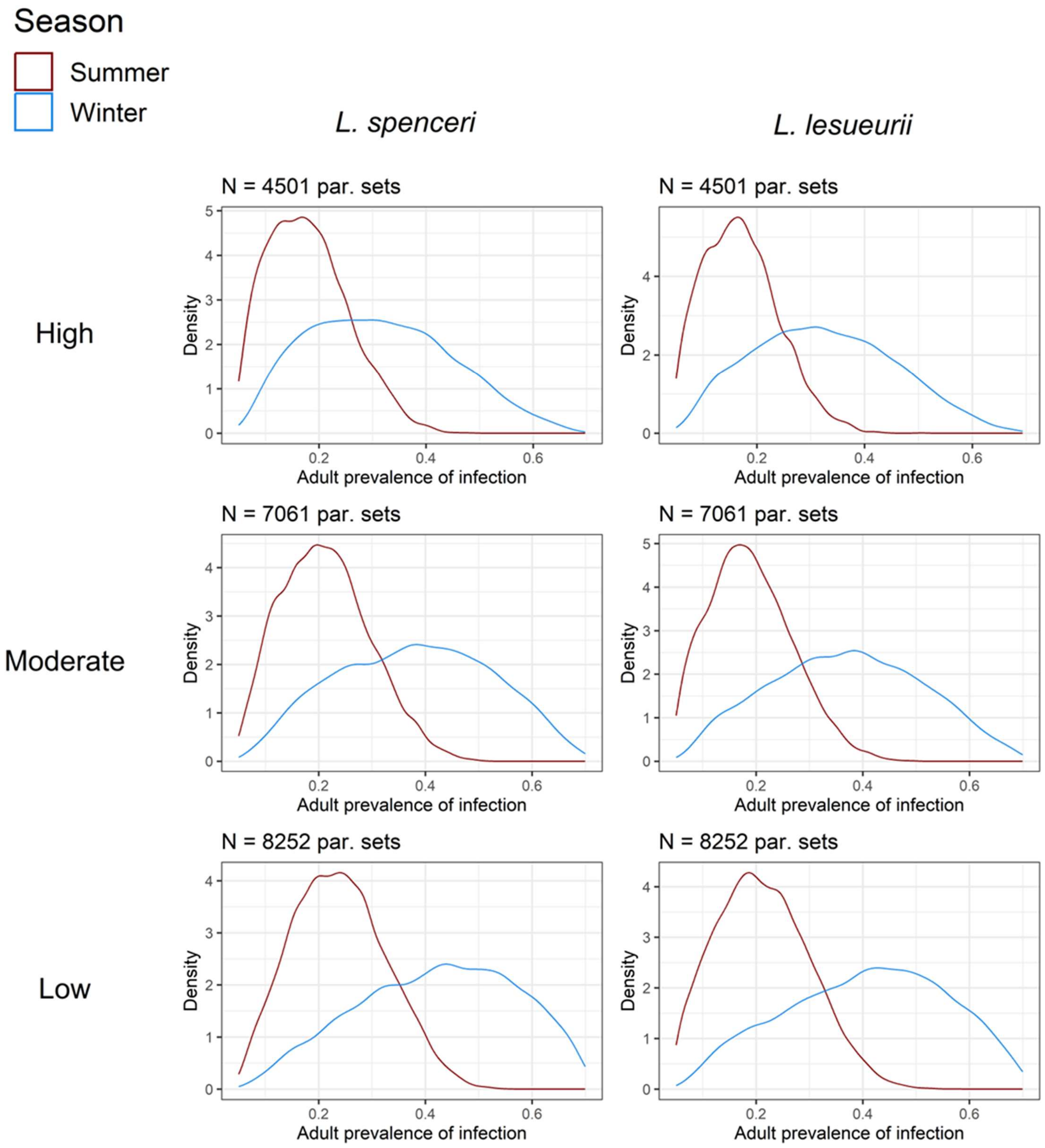

Figure S3. Density curves demonstrating variation in the summer (red) and winter (blue) prevalence of infection in the year of quasi-equilibrium for high, moderate, and low elevation populations of *L. spenceri* and *L. lesueurii* across all quasi-equilibrated parameter sets which passed realism filtering criteria (number of parameter sets listed above each panel). Prevalence of infection was calculated in the adult stage.

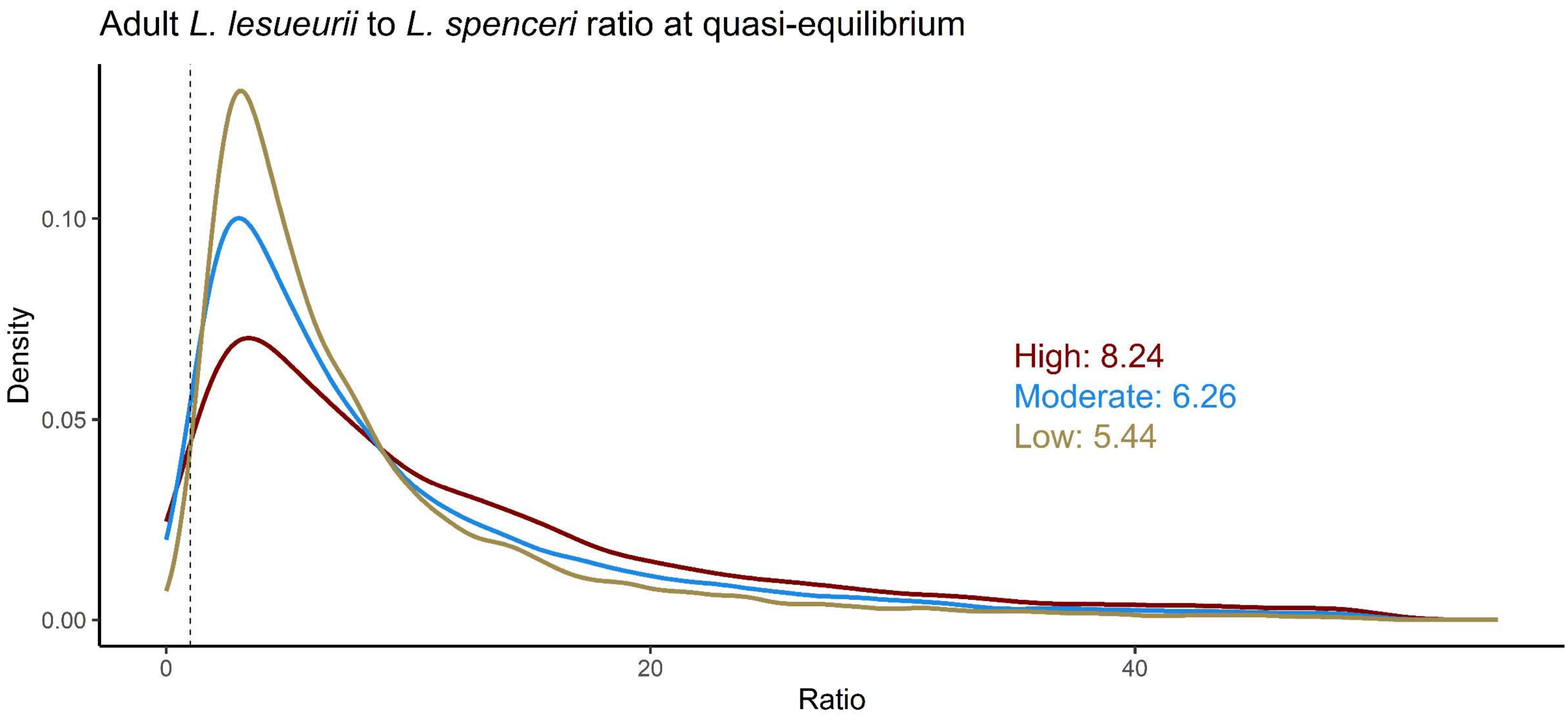

Figure S4. Density curves demonstrating variation in the adult *L. lesueurii*-to-*L. spenceri* ratio at quasi-equilibrium across high elevation (red), moderate elevation (blue), and low elevation (gold) parameter sets which passes realism criteria, where the dotted line indicates a 1:1 ratio between the two species and the median adult *L. lesueurii*-to-*L. spenceri* ratio is listed for each elevation.

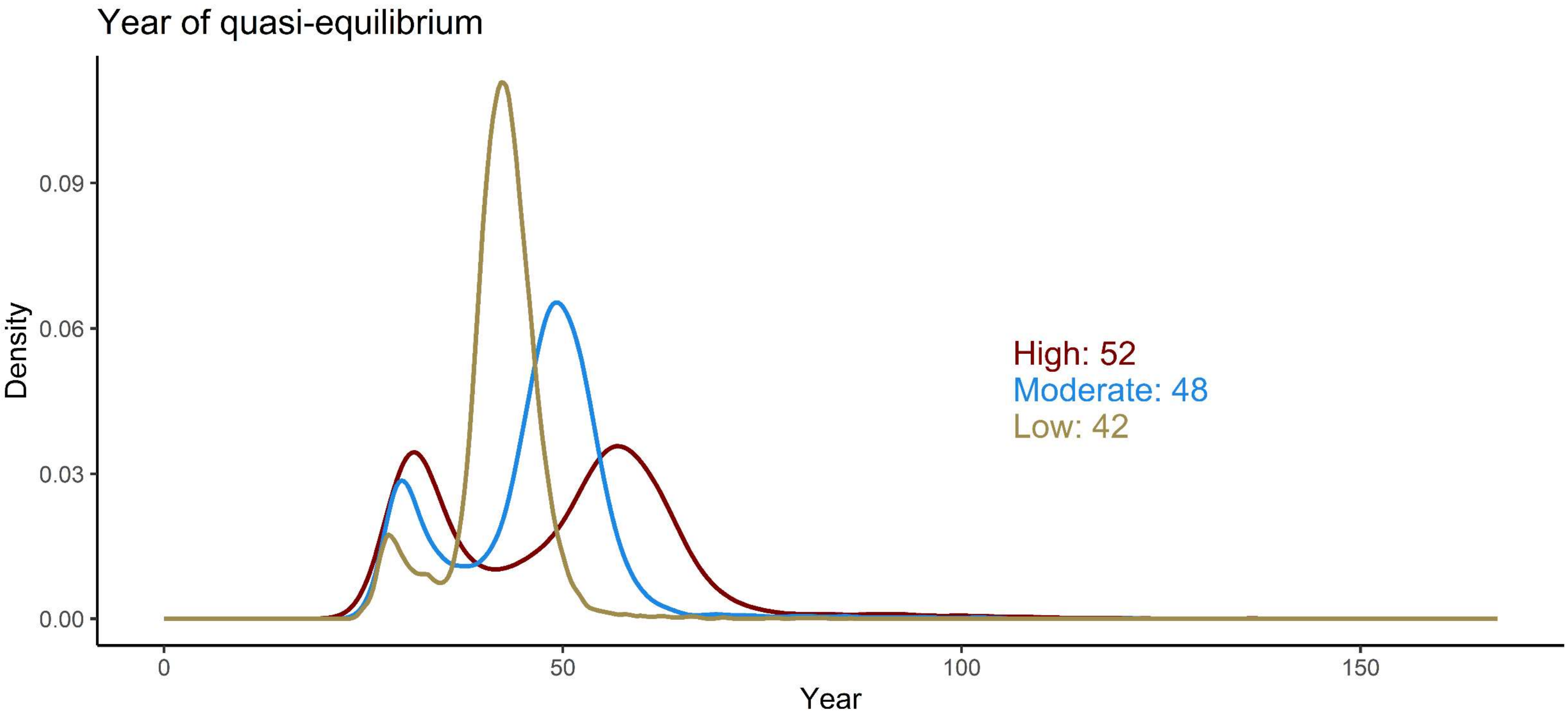

Figure S5. Density curves demonstrating variation in the number of years required to reach quasi-equilibrium across all parameter sets which passed realism criteria for high elevation (red), moderate elevation (blue), and low elevation (gold) models, where the median year of quasi-equilibrium is listed for each elevation. Quasi-equilibrium was defined when both species experienced a <1% change in adult density for 20 consecutive years (but see methods for special case where one species is moving towards extinction).

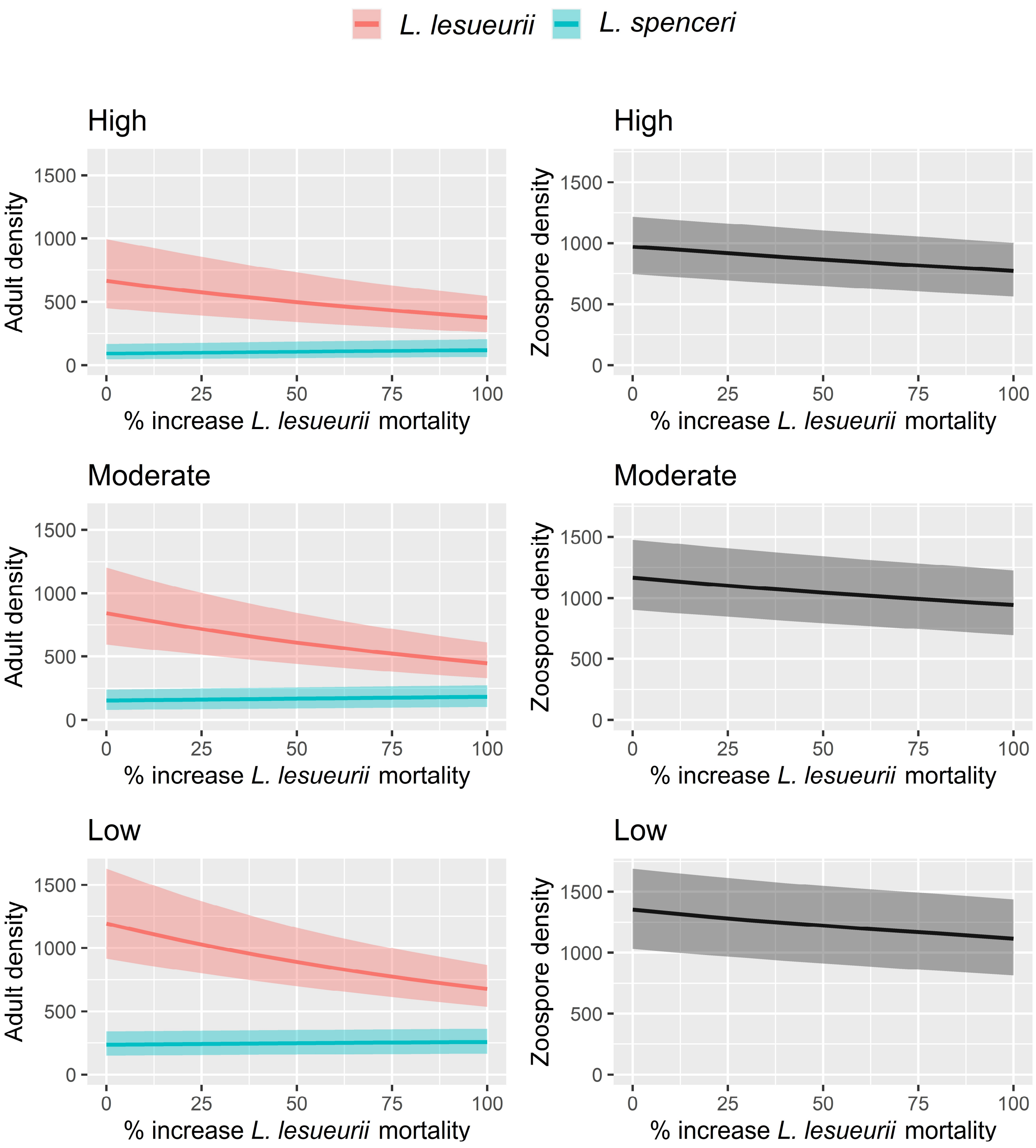

Figure S6. Median model compartment densities at high, moderate, and low elevations after 20-year simulations, across varying degrees of intervention to remove *L. lesueurii* frogs. Bands represent the lower (25%) and upper (75%) quartiles of the final density for adult *L. lesueurii* (red), adult *L. spenceri* (blue), and Bd zoospores (black). Intervention to remove *L. lesueuri*i frogs was simulated by increasing post-metamorphic *L. lesueurii* mortality from its baseline value (0% intervention) to that of *L. spenceri* (100% intervention).

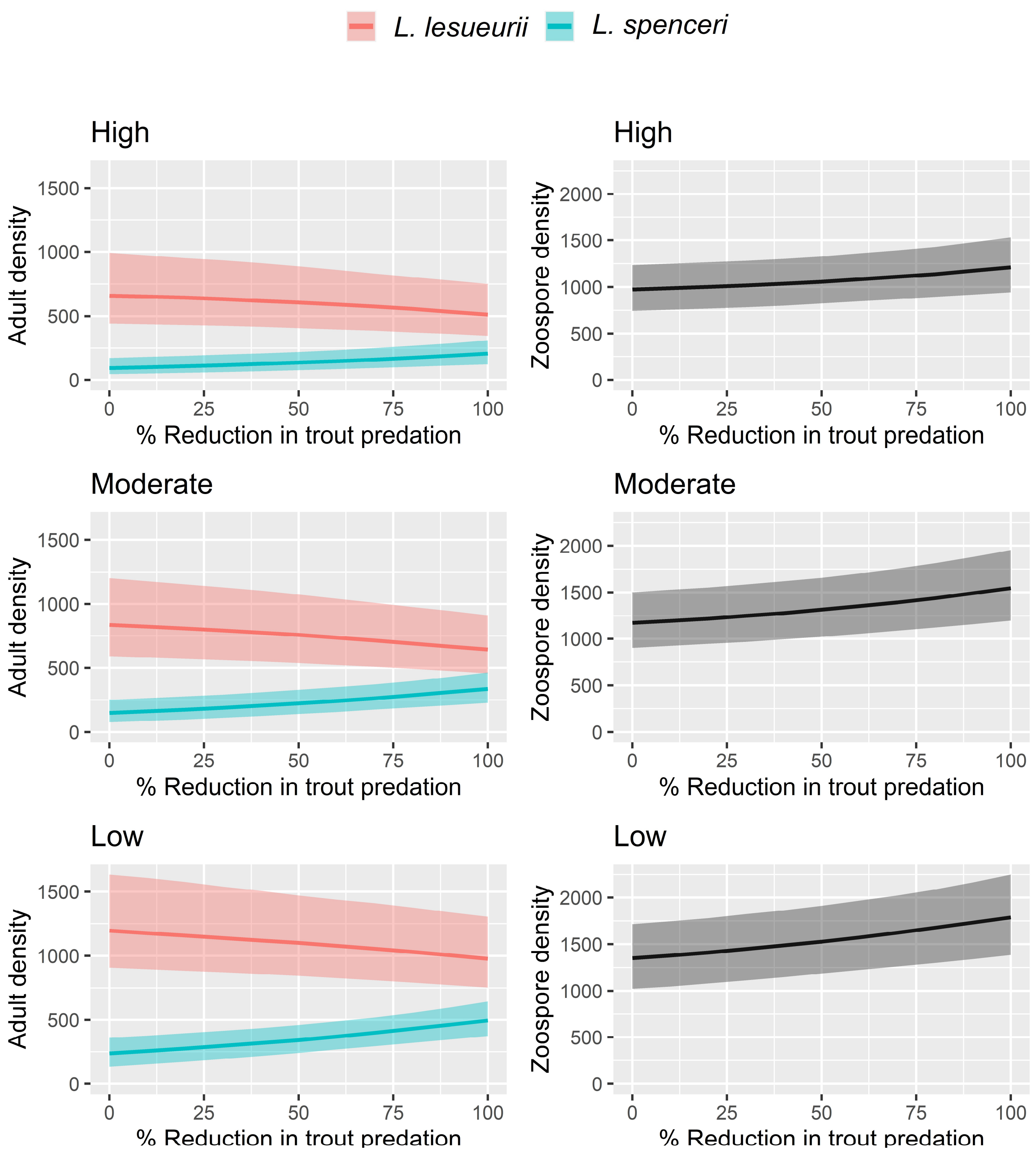

Figure S7. Median model compartment densities at high, moderate, and low elevations after 20-year simulations, across varying degrees of intervention to reduce predation of *L. spenceri* by invasive trout. Bands represent the lower (25%) and upper (75%) quartiles of the final density for adult *L. lesueurii* (red), adult *L. spenceri* (blue), and Bd zoospores (black). Intervention to reduce predation was scaled by reducing *L. spenceri* larval and juvenile mortality from their baseline values (0% intervention) to those of *L. lesueurii* (100% intervention).

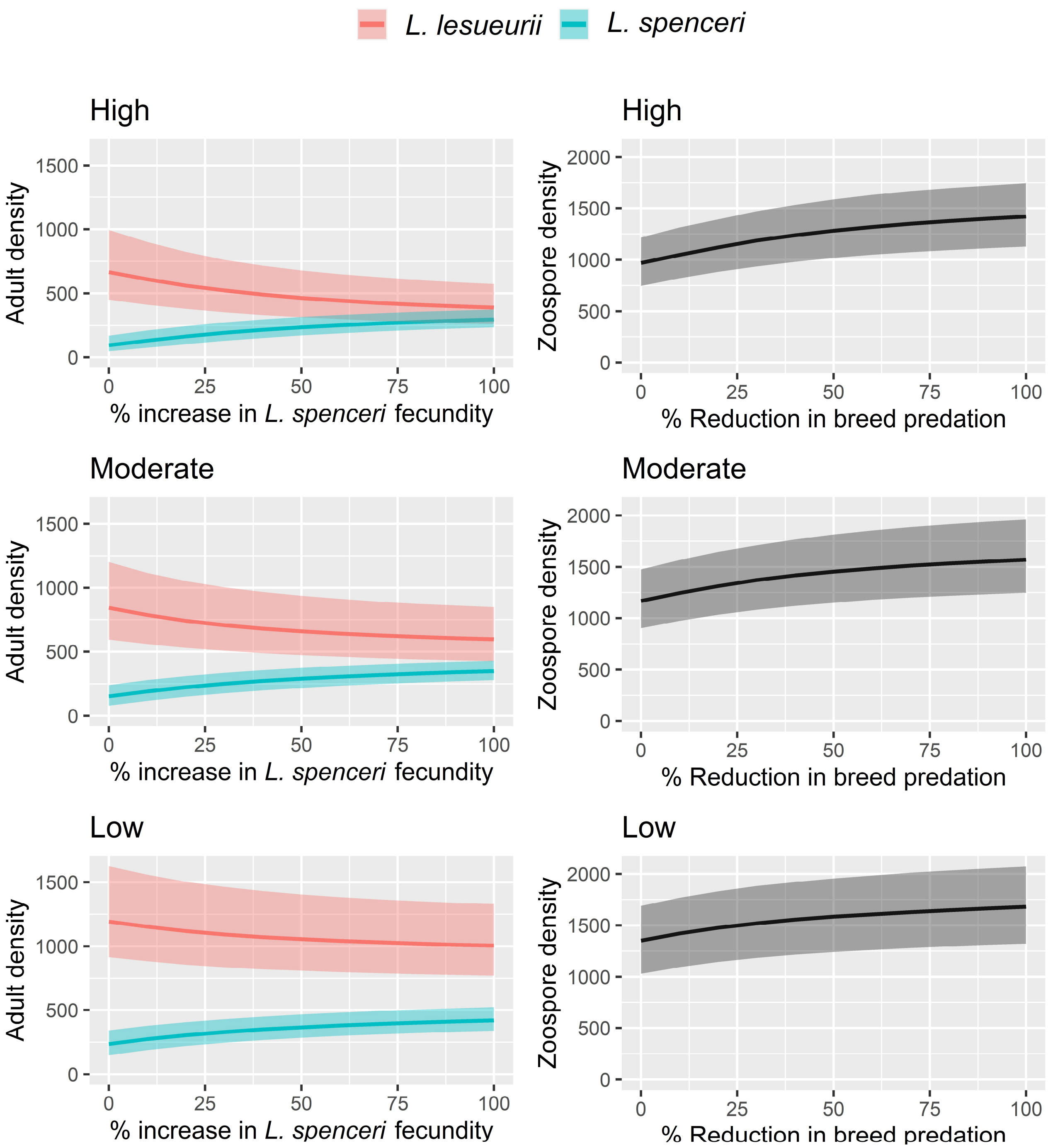

Figure S8. Median model compartment densities at high, moderate, and low elevations after 20-year simulations, across varying degrees of intervention to increase *L. spenceri* fecundity through captive breeding. Bands represent the lower (25%) and upper (75%) quartiles of the final density for adult *L. lesueurii* (red), adult *L. spenceri* (blue), and Bd zoospores (black). Intervention to bolster *L. spenceri* fecundity was scaled from the innate fecundity of *L. spenceri* (0% intervention) until matching the innate fecundity of *L. lesueurii* (100% intervention) for each elevation.

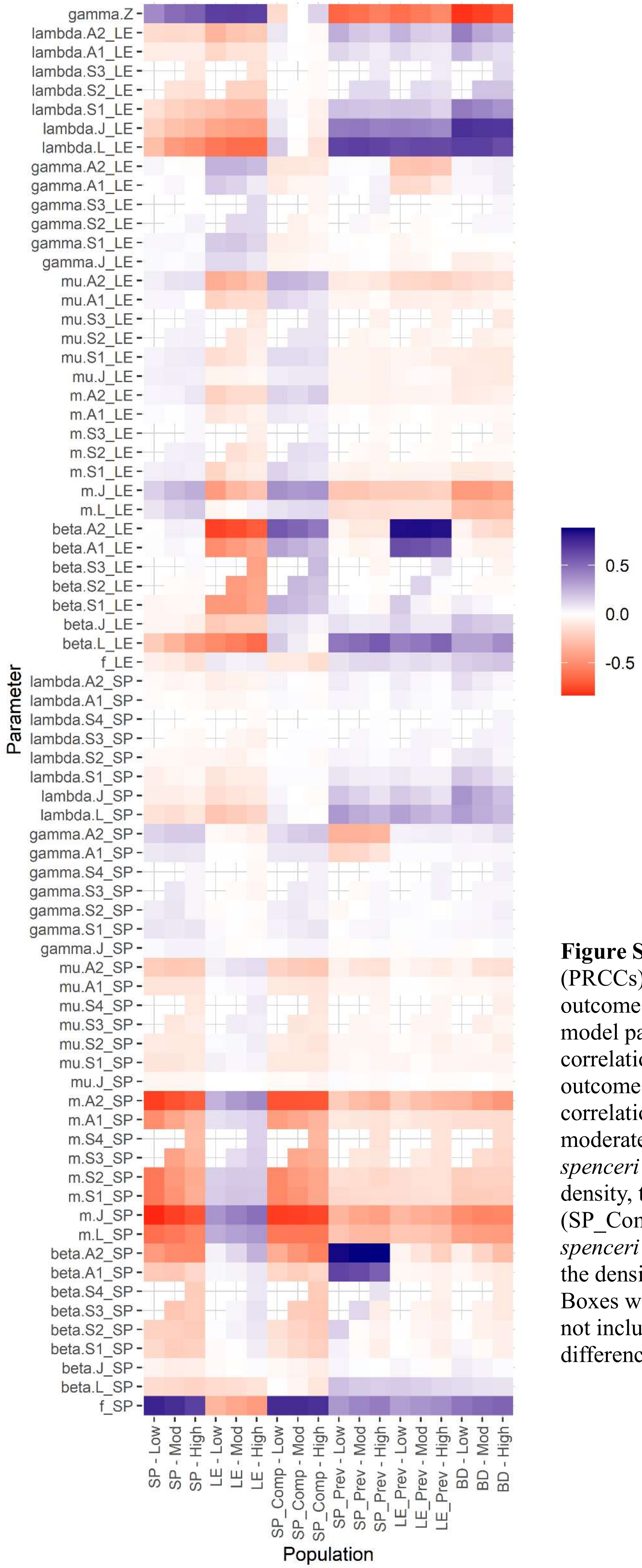

Figure S9. Partial Rank Correlation Coefficients (PRCCs) displaying the sensitivity of population outcomes at quasi-equilibrium to variation in each model parameter. Blue values represent a positive correlation between the parameter and the model outcome, while red values represent a negative correlation. The PRCCs are shown across low (Low), moderate (Mod), and high (High) elevations for *L. spenceri* (SP) adult density, *L. lesueurii* (LE) adult density, the competitive success of *L. spenceri* (SP_Comp), the prevalence of infection for *L. spenceri* (SP_Prev) and *L. lesueurii* (LE_Prev), and the density of zoospores in the environment (BD). Boxes with grey hashes indicate a parameter that was not included in the model due to life history differences across elevations.

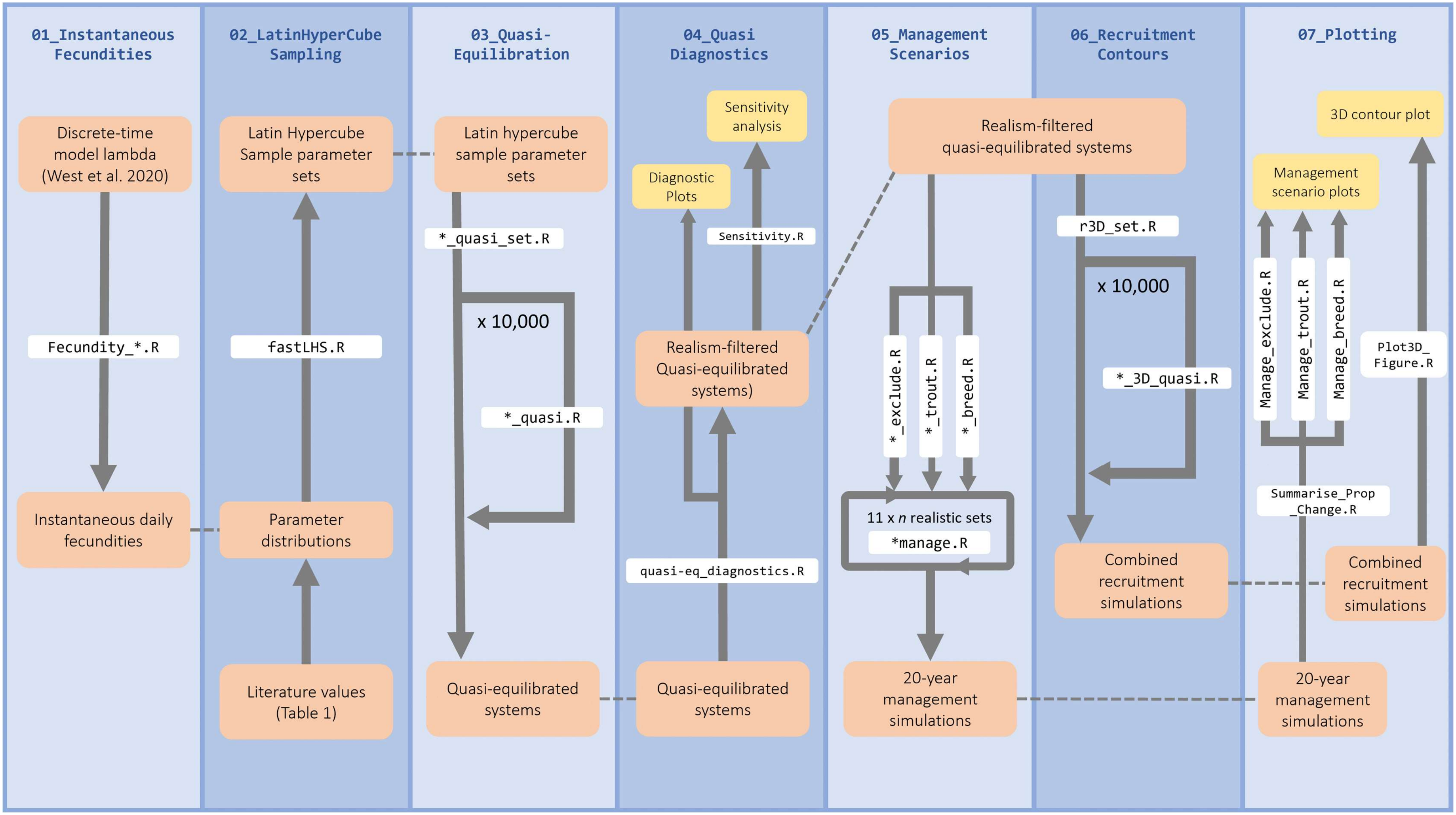

Figure S10. Overview of the SI model R script pipeline to generate quasi-equilibrium diagnostics, a sensitivity analysis, 20-year management outcomes, and combined trout and captive breeding contour plots from prior parameter distributions at each elevation. An asterisk * represents a wildcard placeholder to signify either "high", "moderate" or "low" elevation scripts. Grey arrows and hashed lines indicate the direction of the workflow, and dashed lines indicate the transfer of data files between directories (from left to right).
